# Neuronal APOE4 removal protects against tau-mediated gliosis, neurodegeneration and myelin deficits

**DOI:** 10.1038/s43587-023-00368-3

**Published:** 2023-02-20

**Authors:** Nicole Koutsodendris, Jessica Blumenfeld, Ayushi Agrawal, Michela Traglia, Brian Grone, Misha Zilberter, Oscar Yip, Antara Rao, Maxine R. Nelson, Yanxia Hao, Reuben Thomas, Seo Yeon Yoon, Patrick Arriola, Yadong Huang

**Affiliations:** 1 Gladstone Institute of Neurological Disease, Gladstone Institutes, San Francisco, CA USA; 2 Developmental and Stem Cell Biology Graduate Program, University of California, San Francisco, San Francisco, CA USA; 3 Neuroscience Graduate Program, University of California, San Francisco, San Francisco, CA USA; 4 Gladstone Institute of Data Science and Biotechnology, Gladstone Institutes, San Francisco, CA USA; 5 Gladstone Center for Translational Advancement, Gladstone Institutes, San Francisco, CA USA; 6 Biomedical Sciences Graduate Program, University of California, San Francisco, San Francisco, CA USA; 7 Departments of Neurology and Pathology, University of California, San Francisco, San Francisco, CA USA

**Keywords:** Alzheimer's disease, Neurodegeneration, Ageing

## Abstract

Apolipoprotein E4 (*APOE4*) is the strongest known genetic risk factor for late-onset Alzheimer’s disease (AD). Conditions of stress or injury induce APOE expression within neurons, but the role of neuronal APOE4 in AD pathogenesis is still unclear. Here we report the characterization of neuronal APOE4 effects on AD-related pathologies in an APOE4-expressing tauopathy mouse model. The selective genetic removal of APOE4 from neurons led to a significant reduction in tau pathology, gliosis, neurodegeneration, neuronal hyperexcitability and myelin deficits. Single-nucleus RNA-sequencing revealed that the removal of neuronal APOE4 greatly diminished neurodegenerative disease-associated subpopulations of neurons, oligodendrocytes, astrocytes and microglia whose accumulation correlated to the severity of tau pathology, neurodegeneration and myelin deficits. Thus, neuronal APOE4 plays a central role in promoting the development of major AD pathologies and its removal can mitigate the progressive cellular and tissue alterations occurring in this model of APOE4-driven tauopathy.

## Abstract (web summary)

APOE4 is produced by neurons under stress, but the role of neuronal APOE4 in Alzheimer’s disease pathogenesis is still unclear. Here the authors report that selective removal of neuronal APOE4 in tauopathy mice mitigates many prominent Alzheimer’s disease-related pathologies.

## Main

Tauopathies are a class of neurodegenerative disorders defined by the abnormal intracellular accumulation of hyperphosphorylated tau (p-tau) protein^1–3^. AD is a major type of tauopathy that is characterized by memory loss and the accumulation of amyloid plaques and tau tangles^4,5^. Of these two major AD pathological hallmarks, tau tangles have the strongest correlation with neurodegeneration and cognitive decline^6–9^. Other AD pathological hallmarks that have been understudied include neuroinflammation and gliosis, which have recently been shown to be key drivers of neurodegeneration^10,11^. Additionally, oligodendrocyte deficits and myelin degeneration have been observed in human AD brains^12–14^ and in mouse models of AD and tauopathy^15–17^. Thus, AD is a multifactorial disorder that consists of a complex set of pathologies; however, the connections between these pathologies and the mechanisms responsible for their induction or exacerbation remain unclear.

*APOE4* is the major genetic risk factor for late-onset AD^18–20^. While the human *APOE* gene has three common alleles, 𝜀2, 𝜀3 and 𝜀4, the *APOE* 𝜀4 allele is considered the most detrimental as it leads to an increase in AD risk and a decrease in the age of disease onset^18–21^. There have been great efforts to understand how APOE4 increases AD risk, with an extensive body of work indicating that APOE4 worsens many prominent AD-related pathologies relative to APOE3 (refs. ^22,23^). In particular, APOE4 has been shown to accelerate hippocampal volume loss in human patients^24^ and to increase neurodegeneration in mice^25–28^. APOE4 also increases tau burden in human brains^8,9,29,30^ and promotes the accumulation of p-tau in human neurons^31–33^ and mouse models^28,34,35^. Furthermore, APOE4 increases neuroinflammation and gliosis in human AD brains^36–38^ and in tauopathy mouse models^28^. It has also been reported that APOE4 is associated with reduced myelination and white matter integrity in human brains^39^. Together, these studies show clear evidence that APOE4 is implicated in promoting tau pathology, gliosis, neurodegeneration and myelin degeneration in AD and other tauopathies. Nonetheless, the underlying mechanisms responsible for APOE4’s wide-ranging effects on these various pathologies remain elusive.

Recently, there has been increasing interest in establishing the cell-type-specific effects of APOE4 in AD pathogenesis. Within the central nervous system (CNS), APOE is produced by a variety of cell types and previous studies have indicated that APOE exerts different pathological effects depending on its cellular source^35,40–42^. APOE is mainly produced by astrocytes in the CNS, although conditions of stress or injury induce APOE expression in neurons^27,43,44^. Recent findings have implicated astrocytic APOE4 in the pathogenesis of AD, as genetic deletion of APOE4 in astrocytes led to a reduction of various AD-related pathologies^41^. Still, there is an incomplete understanding of the role of neuronal APOE4 in the pathogenesis of AD. Previous studies from our laboratory have suggested that neuronal APOE4 is important to some AD-related processes, such as tau phosphorylation, inhibitory neuron loss and memory deficits^35,40^; however, it is still unclear whether neuronal APOE4 represents a key pathogenic factor driving the development of full-scale AD pathologies. It is critically important to elucidate the exact role of neuronal APOE4 in the pathogenesis of AD to gain a better understanding of the cellular source-specific mechanisms that drive the detrimental effects of APOE4 and to potentially reveal new therapeutic targets to combat APOE4-related AD.

In the present study, we conducted an extensive analysis of human APOE4- and APOE3-expressing tauopathy mouse models to investigate the impact of selectively removing APOE4 from neurons on the development of major AD pathologies, including tau pathology, gliosis, neurodegeneration, neurodysfunction and myelin deficits. The outcomes of this study should provide new insights into the full-scale roles of neuronal APOE4 in the pathogenesis of AD and other tauopathies.

## Results

### Neuron-specific removal of the *APOE* gene in tauopathy mice

Our laboratory previously generated mouse lines expressing a floxed human *APOE3* or *APOE4* gene^45^ and a Cre recombinase gene under the control of a neuron-specific synapsin-1 promoter (Syn1-Cre)^46^. These floxed *APOE-KI* (fE) mice express homozygous human *APOE3* or *APOE4* in place of the endogenous mouse *Apoe* and the human *APOE* gene is flanked by a pair of LoxP sites to allow for its precise excision in the presence of cell-type-specific Cre recombinase expression^40^. The fE mice with or without Syn1-Cre were crossbred with mice expressing mutant 1N4R human microtubule-associated protein tau (*MAPT*) encoding the disease-associated P301S mutation (PS19 line), which has been widely utilized as a tauopathy mouse model^47^. The resulting compound mice are referred to as PS19-fE or PS19-fE/Syn1-Cre mice.

**Extended Data Fig. 1:**
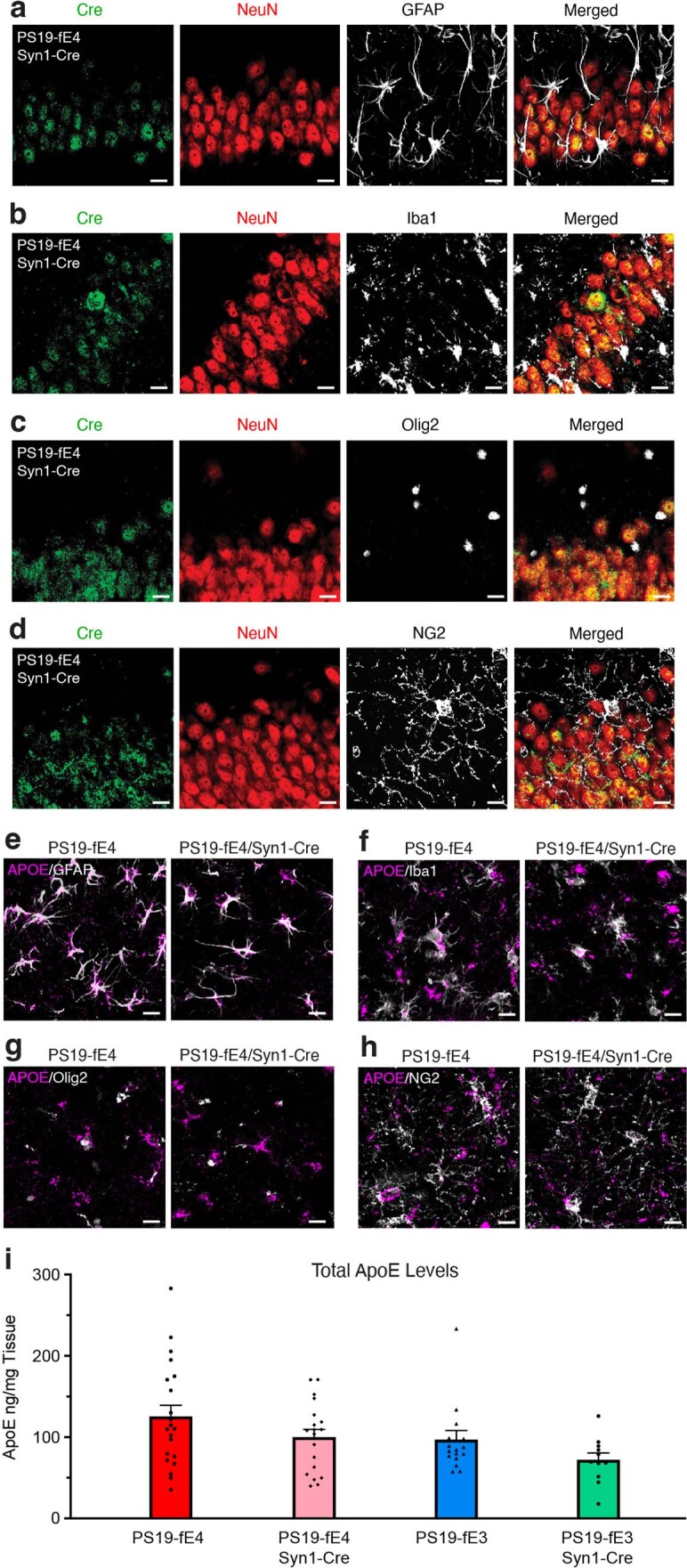
Characterization of PS19-fE mouse models with the *APOE* gene specifically removed from neurons by neuron-specific Syn1-Cre expression. **a–d**, Representative images of the cell type-specificity of Cre recombinase expression in various cell types as determined by immunostaining for Cre recombinase in neurons (NeuN) (a–d), astrocytes (GFAP) (a), microglia (Iba1) (b), oligodendrocytes (Olig2) (c), and OPCs (NG2) (d) in the hippocampus of 10-month-old PS19-fE4 mice with Syn1-Cre (scale bar, 20 µm). **e–h**, Representative images of the cell type-specificity of APOE expression in various cell types by immunostaining for APOE in astrocytes (e), microglia (f), oligodendrocytes (g), and OPCs (h) in the hippocampus of 10-month-old PS19-fE4 mice with and without Cre (scale bar, 20 µm). **i**, APOE levels measured by ELISA in the hippocampal lysates of 10-month-old PS19-fE4 and PS19-fE3 mice with and without Cre (PS19-fE4: No Cre, n = 21; Syn1-Cre, n = 19; PS19-fE3: No Cre, n = 17; Syn1-Cre, n = 11). APOE levels are normalized to the weight of the dissected hippocampal tissue for each individual mouse. Experiments depicted in representative images in a–h were performed on n = 4 mice per genotype using 2 brain sections per mouse, with reproducible data. Data are represented as mean ± SEM, one-way ANOVA with Tukey’s post hoc multiple comparisons test. Source data

We previously performed a rigorous characterization of fE/Syn1-Cre mice to validate the specificity of Cre recombinase expression under the neuron-specific Syn1 promoter^40^. To further confirm its specificity in 10-month-old PS19-fE mice, we immunostained for Cre recombinase in multiple APOE-expressing cell types, including neurons (NeuN), astrocytes (GFAP), microglia (Iba1), oligodendrocytes (Olig2) and oligodendrocyte progenitor cells (OPCs) (NG2). In PS19-fE4/Syn1-Cre mice, Cre recombinase was expressed exclusively in neurons and was not expressed in the other four cell types (Extended Data Fig. 1a–d), which agrees with our previous study showing that Syn1-Cre mice crossbred with a Cre-EGFP-reporter mouse line exhibit expression of Cre-driven EGFP only in neurons in the hippocampus and cortex^40^. Immunostaining for APOE in these relevant cell types showed that both PS19-fE4 and PS19-fE4/Syn1-Cre mice had APOE expression in astrocytes, microglia, oligodendrocytes and OPCs (Extended Data Fig. 1e–h).

To quantitatively determine the levels of human APOE protein in these various mouse models, we analyzed hippocampal lysates of 10-month-old mice by sandwich ELISA. PS19-fE4/Syn1-Cre mice exhibited a ~20% decrease in APOE levels relative to PS19-fE4 mice (Extended Data Fig. 1i), which aligns with our previous reports that neuronal APOE contributes to ~20–30% of total APOE protein levels in the hippocampus and cortex^27,40^. Similarly, PS19-fE3/Syn1-Cre mice exhibited a ~25% decrease in APOE levels relative to PS19-fE3 mice (Extended Data Fig. 1i). These results provide strong evidence that *APOE* gene expression is selectively eliminated in neurons when Cre recombinase expression is driven under a Syn1 promoter, while leaving APOE expression in other relevant APOE-expressing cell types in the brain, which is confirmed by single-nucleus RNA-sequencing (snRNA-seq) analysis (see below).

### Removal of APOE4 from neurons drastically reduces tau pathology

**Fig. 1:**
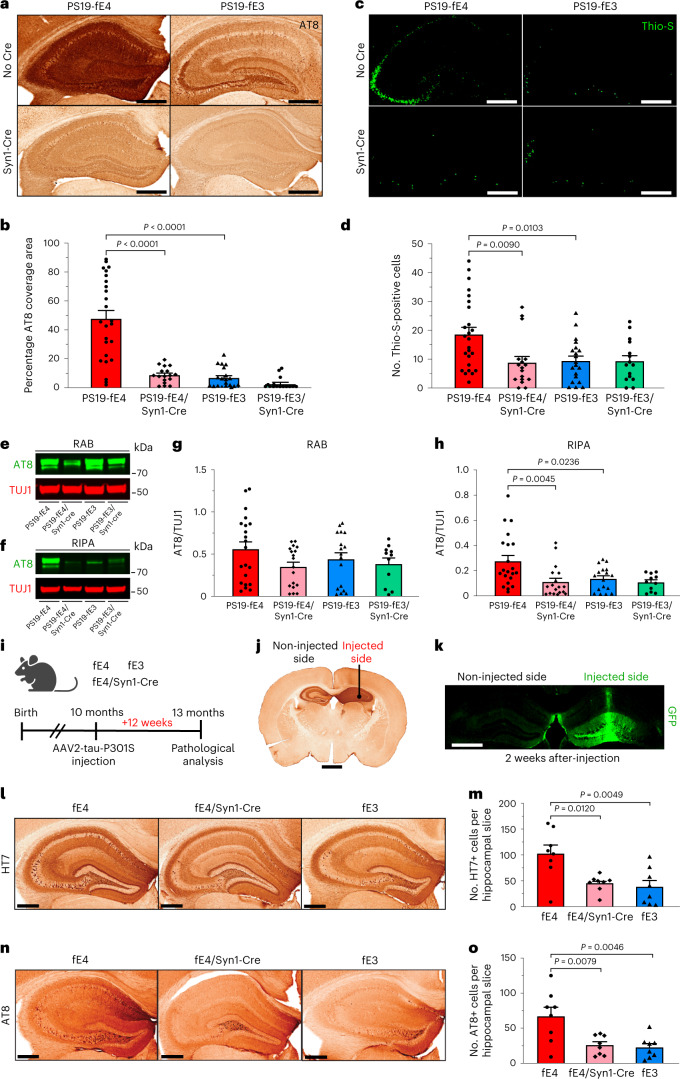
Tau pathology and its spread are significantly reduced after neuronal APOE4 removal. **a**, Representative images of p-tau staining with AT8 antibody in the hippocampus of 10-month-old PS19-fE4 and PS19-fE3 mice with and without Cre (scale bar, 500 µm). **b**, Quantification of the percent p-tau (AT8) coverage area in the hippocampus of 10-month-old PS19-fE4 and PS19-fE3 mice with or without Cre. **c**, Representative images of Thio-S staining in the hippocampus of 10-month-old PS19-fE4 and PS19-fE3 mice with and without Cre (scale bar, 500 µm). **d**, Quantification of the number of Thio-S-positive cells in the hippocampus of 10-month-old PS19-fE4 and PS19-fE3 mice with or without Cre. **e**,**f**, Representative images of anti-p-tau (AT8, green) and anti-TUJ1 (red) western blots in RAB (**e**) and RIPA (**f**) fractions of hippocampal tissue lysates from 10-month-old PS19-fE4 and PS19-fE3 mice with or without Cre. **g**,**h**, Quantification of AT8-positive p-tau levels relative to TUJ1 measured by western blot analysis in RAB (**g**) and RIPA (**h**) fractions of the hippocampal lysates from 10-month-old PS19-fE4 and PS19-fE3 mice with or without Cre. **i**, Experimental design of tau propagation study following unilateral hippocampal injection of AAV2-Tau-P301S in fE mice with and without Cre. **j**, Representative images of human tau immunostaining (HT7) of a fE4 mouse brain 12 weeks after injection, with the injection site indicated by the black dot and a notch to distinguish the non-injected side (scale bar, 1 mm). **k**, Representative image of GFP immunostaining of a 10-month-old fE4 mouse 2 weeks after a unilateral injection with AAV2-GFP (scale bar, 900 µm). **l**, Representative images of human tau immunostaining (HT7) on the non-injected hippocampal side of 13-month-old fE mice with and without Cre (scale bar, 500 µm). **m**, Quantification of the average number of HT7 (human tau)-positive cells in each hippocampal slice on the non-injected hippocampal side of 13-month-old fE mice with or without Cre 12 weeks after injection. **n**, Representative images of p-tau immunostaining (AT8) on the non-injected hippocampal side of 13-month-old fE mice with or without Cre (scale bar, 500 µm). **o**, Quantification of the average number of AT8 (p-tau)-positive cells in each hippocampal slice on the non-injected hippocampal side of 13-month-old fE mice with or without Cre 12 weeks after injection. For quantifications in **b**,**d**, PS19-fE4: No Cre, *n* = 25; Syn1-Cre, *n* = 17; PS19-fE3: No Cre, *n* = 20; Syn1-Cre, *n* = 15. For quantifications in **g**,**h**, PS19-fE4: No Cre, *n* = 21; Syn1-Cre, *n* = 18; PS19-fE3: No Cre, *n* = 17; Syn1-Cre, *n* = 11.Quantified data in **m**,**o** are *n* = 8 mice per genotype and data in **b**,**d**,**g**,**h**,**m**,**o** are represented as mean ± s.e.m., one-way analysis of variance (ANOVA) with Tukey’s post hoc multiple comparisons test. Source data. Source data

To determine whether the removal of neuronal APOE affects tau pathology, we assessed mice at 10 months of age, which is when PS19 mice exhibit extensive tau pathology throughout the hippocampus^47^. We immunostained with the p-tau-specific AT8 antibody. PS19-fE4 mice exhibited significantly more tau pathology throughout the hippocampus than PS19-fE3 mice (Fig. 1a,b). PS19-fE4/Syn1-Cre mice exhibited a notable reduction (~81%) in tau pathology relative to PS19-fE4 mice (Fig. 1a,b). There was no significant difference in tau pathology between PS19-fE3 with and without Cre, likely because the tau pathology in PS19-fE3 mice was already low. Assessment of neurofibrillary tangles by staining with thioflavine S (Thio-S) revealed a similar pattern, with PS19-fE4 mice exhibiting a much higher number of Thio-S-positive cells in the hippocampus than PS19-fE3 mice and removal of neuronal APOE4 leading to a significant decrease in the number of neurofibrillary tangle-bearing cells (Fig. 1c,d).

**Extended Data Fig. 2:**
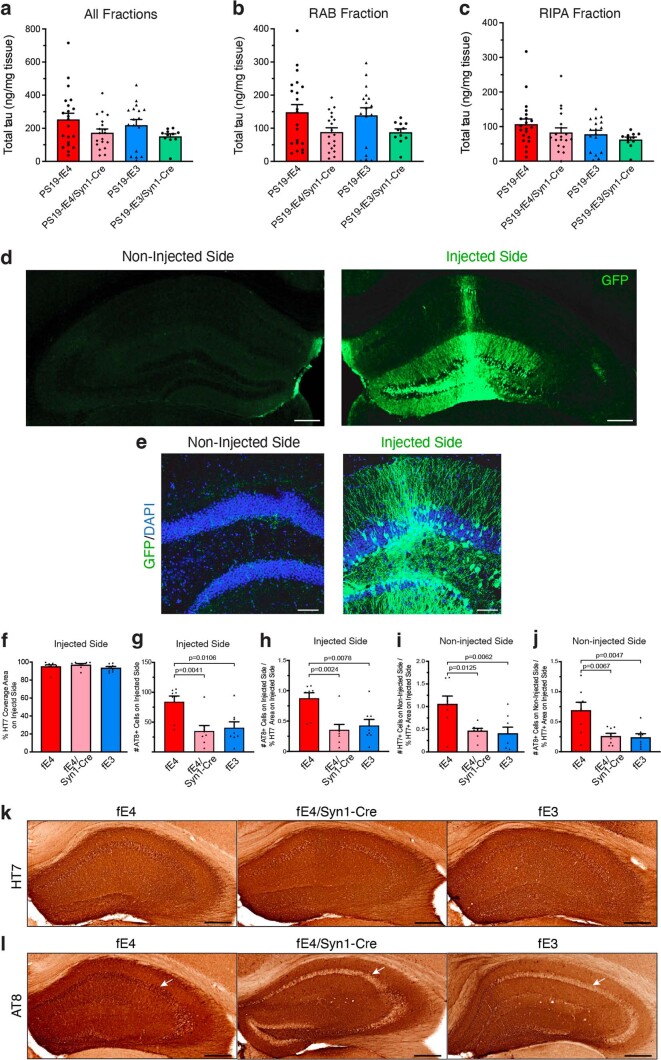
Total tau levels in PS19-fE4 and PS19-fE3 mice with or without Cre and validation of tau spread study. **a–c**, Total tau levels measured by ELISA in the hippocampal lysates of 10-month-old PS19-fE4 and PS19-fE3 mice with and without Cre, following sequential biochemical extraction in RAB and RIPA buffer (PS19-fE4: No Cre, n = 21; Syn1-Cre, n = 19; PS19-fE3: No Cre, n = 17; Syn1-Cre, n = 11). Total tau levels were normalized to the weight of the dissected hippocampal tissue for each individual mouse. **d**, Representative images of the injected and non-injected hippocampal sides of a 10-month-old fE4 mouse 2 weeks after a unilateral injection with an AAV2-GFP virus and immunostained with anti-GFP (scale bar, 500 µm). **e**, Representative high magnification images of the injected and non-injected hippocampal sides of a fE4 mouse 2 weeks after an unilateral injection and immunostained with anti-GFP and DAPI (scale bar, 100 µm). **f**, Quantification of percent HT7 (human tau) coverage area on the injected hippocampal side. **g**, Quantification of number of AT8-positive neurons in the neuronal cell layer on the injected hippocampal side. **h**, Normalization of the number of AT8-positive neurons on the injected hippocampal side to the percent HT7 coverage area on the injected hippocampal side. **i**, Normalization of the number of HT7-positive neurons on the non-injected hippocampal side normalized to the percent HT7 coverage area of the injected hippocampal side. **j**, Normalization of the number of AT8-positive neurons on the non-injected hippocampal side to the percent HT7 coverage area of the injected hippocampal side. Mice used in f–j are 13-month-old fE4, fE4/Syn1-Cre, and fE3 mice (n = 8 per group). **k**, Representative images of HT7 immunostaining on the injected hippocampal side of 13-month-old fE mice with or without Cre (scale bar, 500 µm). **l**, Representative images of AT8 immunostaining on the injected hippocampal side of 13-month-old fE mice with or without Cre (scale bar, 500 µm). White arrows in l indicate neuronal soma areas in hippocampi. Data in a,b,c,f,g,h,i,j are represented as mean ± SEM, one-way ANOVA with Tukey’s post hoc multiple comparisons test. Source data

We utilized western blot to assess levels of AT8^+^ p-tau in mouse hippocampal tissues following sequential biochemical extraction with RAB and RIPA buffers, containing highly soluble and less-soluble tau proteins, respectively^28,48^. There was no significant difference in p-tau levels between the various genotype groups in the RAB fraction, although removal of neuronal APOE4 led to a minor decrease (Fig. 1e,g); however, PS19-fE4/Syn1-Cre and PS19-fE3 mice exhibited a significant reduction in p-tau levels in the RIPA fraction relative to PS19-fE4 mice (Fig. 1f,h). We also evaluated the levels of total tau utilizing ELISA analysis and found no significant difference in total tau levels in the RAB or RIPA fractions among genotypes (Extended Data Fig. 2a–c). This suggests that neuronal APOE4 likely impacts tau through pathogenic mechanisms, such as by promoting its phosphorylation, aggregation and spread (see below), rather than affecting the overall production of the tau protein. Taken together, these data indicate that neuronal APOE4 is a strong driver of tau pathology.

### Propagation of tau pathology is reduced after removal of neuronal APOE4

To investigate the mechanisms by which neuronal APOE4 drives tau pathology, we determined the effects of neuronal APOE4 expression on the propagation of tau. In AD and other tauopathies, pathological tau has been shown to spread between neurons and across interconnected brain regions^49,50^. Previous studies have examined tau propagation in vivo by injecting pathological tau protein directly into mouse brains and showing that tau can spread from its injection site to anatomically connected brain regions^51–55^. To study tau propagation, we injected an adeno-associated virus-2 (AAV2) encoding human P301S mutant tau (AAV2-tau-P301S) into the right dorsal hippocampus of 10-month-old fE mice with or without Syn1-Cre and analyzed the extent of tau propagation from the injected to the non-injected side of hippocampus 12 weeks after injection (Fig. 1i,j). The fE mice utilized for this experiment express the endogenous mouse *Mapt* gene and exhibit minimal tau pathology.

To provide evidence that we are observing the spread of pathological human tau between neurons as opposed to the tau-encoding virus itself traveling to the non-injected side, we tested the unilateral injection of an AAV2 that encodes green fluorescent protein (GFP) (AAV2-GFP) into the right hippocampus of a 10-month-old fE4 mouse. Immunostaining with anti-GFP 2 weeks after injection revealed that the GFP signal remains localized to neurons within the injected hippocampal side (Fig. 1k and Extended Data Fig. 2d,e). The non-injected hippocampal side did not have any evident GFP signal in neuronal somas, although there were some GFP-positive neuronal projections, likely stemming from neurons residing on the injected hippocampal side. This illustrates that the AAV2 itself does not spread between hippocampi following unilateral injection. Furthermore, we quantified the number of soma-positive-tau-containing neurons in the non-injected side to more accurately reflect tau spread between neurons and exclude confounding factors, such as tau-positive commissural fibers from neurons originating from the injected side.

Immunohistochemical staining with a human tau antibody (HT7) on the injected hippocampal side confirmed that mice with different genotypes expressed similar levels of tau-P301S from the injected virus (Extended Data Fig. 2f,k). Immunostaining for p-tau with the AT8 antibody on the injected hippocampal side showed that fE4/Syn1-Cre and fE3 mice have significantly fewer neurons positive for AT8 than fE4 mice (Extended Data Fig. 2g,h,l), which is in line with the observed reduction of p-tau coverage in PS19-fE4/Syn1-Cre mice versus PS19-fE4 mice (Fig. 1a,b).

Immunostaining for HT7 revealed robust human tau propagation to the non-injected hippocampal side in fE4 mice and minimal tau propagation in fE3 mice (Fig. 1l,m), indicating that APOE4 promotes tau spreading. Intriguingly, fE4/Syn1-Cre mice had a significant reduction in human tau propagation to the non-injected hippocampal side relative to fE4 mice (Fig. 1l,m). AT8 immunostaining showed that fE4 mice exhibited robust propagation of p-tau to the non-injected hippocampal side, while fE4/Syn1-Cre and fE3 mice had drastically reduced p-tau propagation (Fig. 1n,o). Normalization of the extent of the propagated tau pathology in the non-injected side to the percent HT7 coverage area on the injected side still showed significant differences between fE4 mice and fE4/Syn1-Cre or fE3 mice (Extended Data Fig. 2i,j). Taken together, these data indicate that one mechanism by which neuronal APOE4 drives tau pathology is by stimulating the propagation of tau and/or p-tau between anatomically connected brain regions.

### Neurodegeneration is reduced after removal of neuronal APOE4

**Fig. 2:**
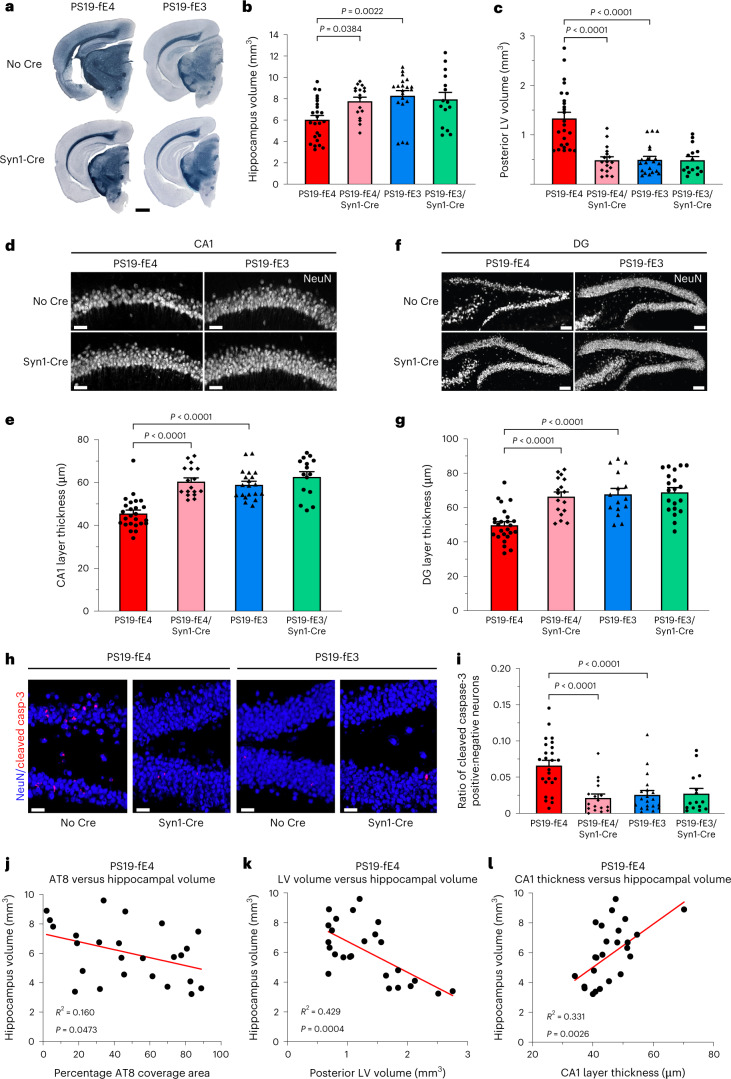
Neurodegeneration is significantly reduced after neuronal APOE4 removal. **a**, Representative images of the ventral hippocampus of 10-month-old PS19-fE4 and PS19-fE3 mice with and without Cre after staining with Sudan Black to enhance hippocampal visualization (scale bar, 1 mm). **b**,**c**, Quantification of hippocampal volume (**b**) and posterior lateral ventricle volume (**c**) in 10-month-old PS19-fE4 and PS19-fE3 mice with and without Cre. **d**, Representative images of the CA1 hippocampal subfield of 10-month-old PS19-fE4 and PS19-fE3 mice with and without Cre after immunostaining for neuronal marker NeuN (scale bar, 50 µm). **e**, Quantification of the thickness of the CA1 neuronal cell layer of 10-month-old PS19-fE4 and PS19-fE3 mice with and without Cre. **f**, Representative images of the hippocampal dentate gyrus of 10-month-old PS19-fE4 and PS19-fE3 mice with and without Cre after immunostaining for neuronal marker NeuN (scale bar, 100 µm). **g**, Quantification of the thickness of the dentate gyrus granule cell layer of 10-month-old PS19-fE4 and PS19-fE3 mice with and without Cre. **h**, Representative images of the hippocampal dentate gyrus of 10-month-old PS19-fE4 and PS19-fE3 mice with and without Cre after immunostaining for NeuN and cleaved caspase-3 (scale bar, 50 µm). **i**, Quantification of the ratio of neurons positive:negative for cleaved caspase-3 in the dentate gyrus of 10-month-old PS19-fE4 and PS19-fE3 mice with and without Cre. For all quantifications in **b**,**c**,**e**,**g**,**i**, PS19-fE4: No Cre, *n* = 25; Syn1-Cre, *n* = 17; and PS19-fE3: No Cre, *n* = 20; Syn1-Cre, *n* = 15 mice. All data are represented as mean ± s.e.m., one-way ANOVA with Tukey’s post hoc multiple comparisons test. **j**–**l**, Correlations between hippocampal volume (mm^3^) and AT8 coverage area (%) (**j**), posterior lateral ventricle volume (mm^3^) (**k**) and CA1 neuronal cell layer thickness (µm) (**l**) in PS19-fE4 mice (*n* = 25). Pearson’s correlation analysis (two-sided). LV, lateral ventricle; DG, dentate gyrus.Source data. Source data

Next, we evaluated the extent of neurodegeneration in 10-month-old PS19-fE mice after the removal of APOE from neurons. Analyses of hippocampal and posterior lateral ventricle volumes revealed that PS19-fE4 mice exhibited extensive neurodegeneration relative to PS19-fE3 mice (Fig. 2a–c). Neurodegeneration was significantly reduced in PS19-fE4/Syn1-Cre mice, while the removal of neuronal APOE3 did not significantly impact neurodegeneration (Fig. 2a–c). Quantification of neuronal cell loss within the various subfields of the hippocampus revealed that PS19-fE4 mice had extensive neuron loss in the hippocampal cornu ammonis 1 (CA1) region and the dentate gyrus and this was significantly reduced in PS19-fE4/Syn1-Cre mice (Fig. 2d–g). There was no significant reduction in neuronal loss after removal of neuronal APOE3 (Fig. 2d–g).

Co-immunostaining for cleaved caspase-3 and NeuN was used to detect postmitotic neurons undergoing apoptosis in the hippocampus. As there were considerable differences in neuronal loss between genotypes (Fig. 2d–g), we quantified the ratio of cleaved caspase-3 positive to negative neurons to normalize for neuronal cell numbers. PS19-fE4 mice had a much higher proportion of neurons positive for cleaved caspase-3 than PS19-fE3 mice and this was significantly reduced after neuronal APOE4 removal (Fig. 2h,i), suggesting that neuronal APOE4 promotes apoptosis of postmitotic neurons in the context of tauopathy.

In PS19-fE4 mice, there was a weak, but significant, negative correlation between tau pathology and hippocampal volume (Fig. 2j), suggesting that tau pathology contributes to the neurodegeneration occurring in these mice. Hippocampal volume also had a strong negative correlation with the posterior lateral ventricle volume and strong positive correlation with the thickness of CA1 (Fig. 2k,l). Taken together, these data illustrate that removal of neuronal APOE4 protects against tau-mediated neurodegeneration and mitigates loss of neurons and hippocampal volume.

### Myelin and oligodendrocyte deficits are reduced after removal of neuronal APOE4

**Fig. 3:**
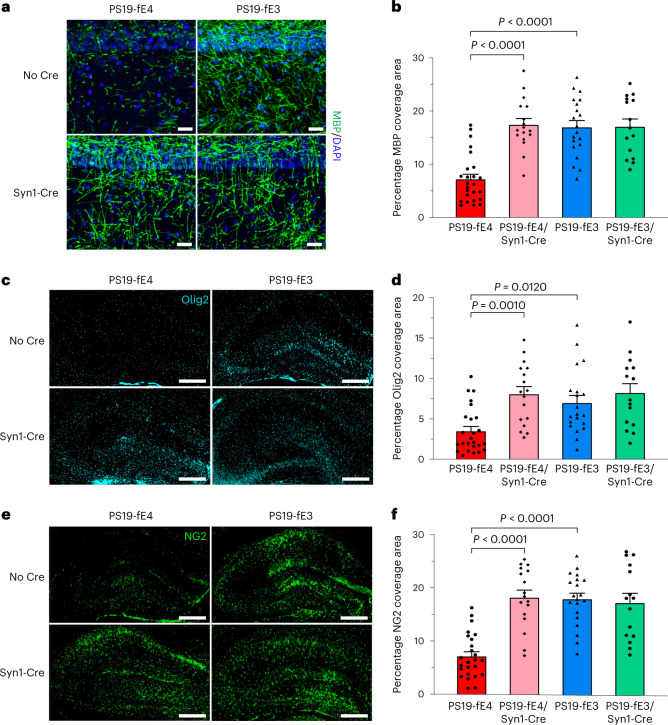
Myelin deficits and depletion of oligodendrocytes and OPCs are significantly reduced after neuronal APOE4 removal. **a**, Representative images of myelin sheath staining with anti-MBP and DAPI in the stratum radiatum of the hippocampus underneath the pyramidal cell layer of CA1 in 10-month-old PS19-fE4 and PS19-fE3 mice with and without Cre (scale bar, 50 µm). DAPI, 4,6-diamidino-2-phenylindole. **b**, Quantification of the percent MBP coverage area in the hippocampal CA1 subregion of 10-month-old PS19-fE4 and PS19-fE3 mice with and without Cre. **c**, Representative images of mature oligodendrocytes by immunostaining with anti-Olig2 in the hippocampus of 10-month-old PS19-fE4 and PS19-fE3 mice with and without Cre (scale bar, 500 µm). **d**, Quantification of the percent Olig2 coverage area in the hippocampus of 10-month-old PS19-fE4 and PS19-fE3 mice with and without Cre. **e**, Representative images of OPCs by immunostaining with anti-NG2 in the hippocampus of 10-month-old PS19-fE4 and PS19-fE3 mice with and without Cre (scale bar, 500 µm). **f**, Quantification of the percent NG2 coverage area in the hippocampus of 10-month-old PS19-fE4 and PS19-fE3 mice with and without Cre. For all quantifications in **b**,**d**,**f**, PS19-fE4: No Cre, *n* = 25; Syn1-Cre, *n* = 17; and PS19-fE3: No Cre, *n* = 20; Syn1-Cre, *n* = 15 mice. All data are represented as mean ± s.e.m., one-way ANOVA with Tukey’s post hoc multiple comparisons test.Source data. Source data

We next investigated the effects of neuronal APOE4 removal on the maintenance of myelin integrity and the density of mature oligodendrocytes in the hippocampus. We immunostained for myelin basic protein (MBP) and quantified the percent coverage area of MBP in the stratum radiatum underneath the pyramidal cell layer of CA1. PS19-fE4 mice had extensive myelin loss relative to PS19-fE3 mice and there was a significant rescue of myelin loss in PS19-fE4/Syn1-Cre mice (Fig. 3a,b). Immunostaining for mature oligodendrocytes with anti-Olig2 showed that PS19-fE4 mice had a lower coverage area of oligodendrocytes in the hippocampus relative to PS19-fE3 mice and the removal of neuronal APOE4 significantly increased the coverage area of mature oligodendrocytes (Fig. 3c,d).

**Extended Data Fig. 3:**
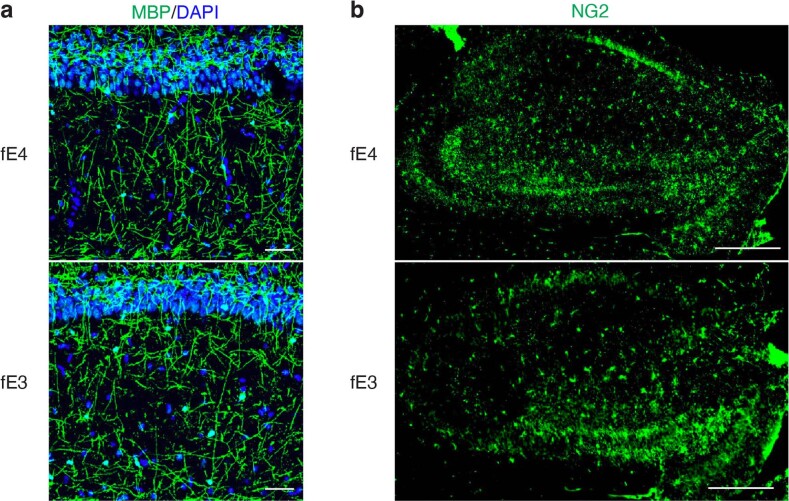
No observable difference in MBP or NG2 immunostaining between fE4 and fE3 mice in the absence of human mutant tau. **a**, Representative images of myelin sheath staining with anti-MBP and DAPI in the stratum radiatum underneath the pyramidal cell layer of CA1 of the hippocampus in 10-month-old fE4 and fE3 mice (scale bar, 50 µm). **b**, Representative images of oligodendrocyte progenitor cell (OPC) staining with anti-NG2 in the hippocampus of 10-month-old fE4 and fE3 mice (scale bar, 500 µm). Experiments depicted in representative images in a–b were performed on n = 4 mice per genotype using 2 brain sections per mouse, with reproducible data.

We also immunostained with an NG2 antibody to probe for OPCs, which have been suggested to aid the repair of damaged myelin in conditions of CNS injury and neurodegeneration^17,56^. We observed a significant decrease in the percent OPC coverage area in the hippocampus of PS19-fE4 mice as compared to PS19-fE3 mice and removal of neuronal APOE4 significantly increased the OPC coverage area (Fig. 3e,f). Immunostaining for myelin and OPCs in fE mice at a similar age revealed that APOE4 mice lacking the human mutant tau-P301S do not exhibit myelin deficits and have similar hippocampal OPC levels as fE3 mice, illustrating that the effects of APOE4 on these phenotypes are dependent on the setting of tauopathy (Extended Data Fig. 3a,b). Overall, these findings suggest that neuronal APOE4 plays a pivotal role in depleting the hippocampal oligodendrocyte and OPC pools and causing myelin deficits in this compound tauopathy mouse model.

### Neuronal network hyperexcitability is eliminated after removal of neuronal APOE4

**Fig. 4:**
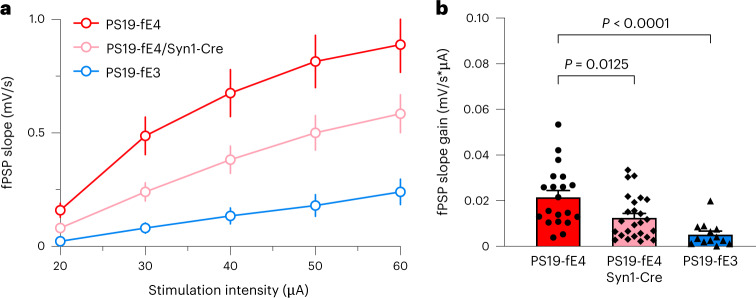
Neuronal APOE4 removal ameliorates neuronal hyperexcitability in the hippocampus. **a**, Average normalized fPSP slopes in CA1 stratum radiatum in response to incremental stimulation of Schaffer collaterals. APOE4 expression renders CA3-CA1 network hyperexcitable as evidenced by augmented response to synaptic stimulation. **b**, Calculated individual field post-synaptic potentials (fPSP) slope gain values for all experiments in **a**. Note that removal of APOE4 from neurons ameliorates neuronal hyperexcitability in the hippocampus of PS19-fE4 mice. PS19-fE4: *n* = 20, *N* = 4; PS19-fE4/Syn1-Cre: *n* = 25, *N* = 4; PS19-fE3: *n* = 13, *N* = 2. Data are represented as mean ± s.e.m., one-way ANOVA with Tukey’s post hoc multiple comparisons test.Source data. Source data

To determine the effects of neuronal APOE4 on neuronal function in the context of tauopathy, we measured neuronal network excitability in the hippocampal CA1 region of PS19-fE3 and PS19-fE4 mice with or without Syn1-Cre by input-output gain analysis of network response to incremental stimulation of Schaffer collaterals^57^ (Fig. 4a). PS19-fE4 mice had notable CA3-CA1 network hyperexcitability as compared to PS19-fE3 mice (Fig. 4b). Removing neuronal APOE4 eliminated neuronal network hyperexcitability (Fig. 4b), indicating that neuronal APOE4 drives neuronal dysfunction in the context of tauopathy.

### Removal of neuronal APOE4 diminishes disease-associated neurons and oligodendrocytes

**Fig. 5:**
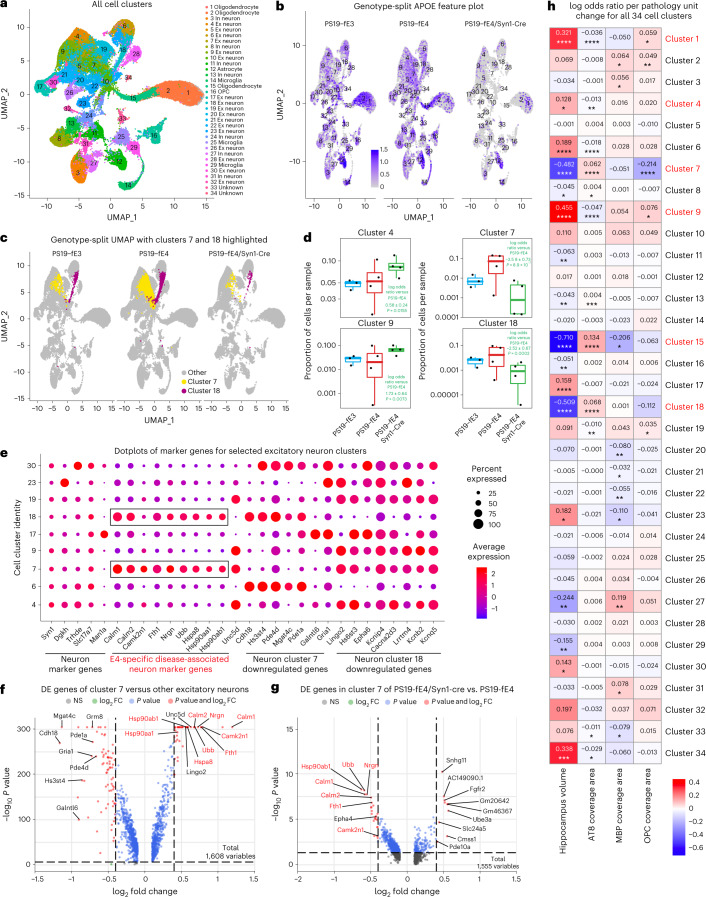
Neuronal APOE4 removal diminishes disease-associated subpopulations of neurons. **a**, UMAP plot of all 34 distinct cell clusters in the hippocampi of 10-month-old PS19-fE4 mice with no Cre (*n* = 4) or with Syn1-Cre (*n* = 4) and PS19-fE3 mice with no Cre (*n* = 3). **b**, Feature plot illustrating the relative levels of normalized human *APOE* gene expression across all 34 hippocampal cell clusters. **c**, UMAP plot highlighting cells in excitatory neuron clusters 7 and 18 for each mouse genotype group. **d**, Box plot of the proportion of cells from each sample in clusters 4, 7, 9 and 18. PS19-fE4 mice with no Cre (*n* = 4) or with Syn1-Cre (*n* = 4) and PS19-fE3 mice with no Cre (*n* = 3). The lower, middle and upper hinges of the box plots correspond to the 25th, 50th and 75th percentiles, respectively. The upper whisker of the box plot extends from the upper hinge to the largest value no further than 1.5 × IQR from the upper hinge. IQR, interquartile range or distance between 25th and 75th percentiles. The lower whisker extends from the lower hinge to the smallest value at most 1.5 × IQR from the lower hinge. Data beyond the end of the whiskers are outlier points. The log odds ratios are the mean ± s.e.m. estimates of log odds ratio for these clusters, which represents the change in the log odds of cells per sample from PS19-fE4/Syn1-Cre mice belonging to the respective clusters compared to the log odds of cells per sample from PS19-fE4 mice. See Supplementary Table 1 for detailed information. **e**, Dot-plot of normalized average expression of marker genes for selected excitatory neuron clusters, highlighting genes that are significantly upregulated and downregulated in excitatory neuron clusters 7 and 18. The size of the dots is proportional to the percentage of cells expressing a given gene. **f**, Volcano plot of the DE genes between excitatory neuron cluster 7 and all other excitatory neuron clusters. Dashed lines represent log_2_ fold change threshold of 0.4 and *P* value threshold of 0.00001. NS, not significant. **g**, Volcano plot of the DE genes in excitatory neuron cluster 7 in PS19-fE4/Syn1-Cre mice versus PS19-fE4 mice. Dashed lines represent log_2_ fold change threshold of 0.4 and *P* value threshold of 0.05. **h**, Heat map plot of the log odds ratio per unit change in each pathological parameter for all cell clusters, with clusters that have significantly increased or decreased logs odds ratio after neuronal APOE4 removal highlighted in red (refer to **d**). The log odds ratio represents the mean estimate of the change in the log odds of cells per sample from a given animal model, corresponding to a unit change in a given histopathological parameter. Negative associations are shown in blue and positive associations are shown in red. Unadjusted *P* values in **d** are from fits to a GLMM_AM and unadjusted *P* values in **h** are from fits to a GLMM_histopathology (Supplementary Table 2, which includes FDR-adjusted *P* values, and Methods provide more details); the associated tests were two-sided. For data in **f**,**g**, the unadjusted *P* values and log_2_ fold change values used were generated from the gene-set enrichment analysis (Methods) using the two-sided Wilcoxon rank-sum test as implemented in the FindMarkers function of the Seurat package. Gene names highlighted in red text indicate they are selected marker genes for DANs. All error bars represent s.e.m. Ex neuron, excitatory neuron; In neuron, inhibitory neuron.

**Extended Data Fig. 4:**
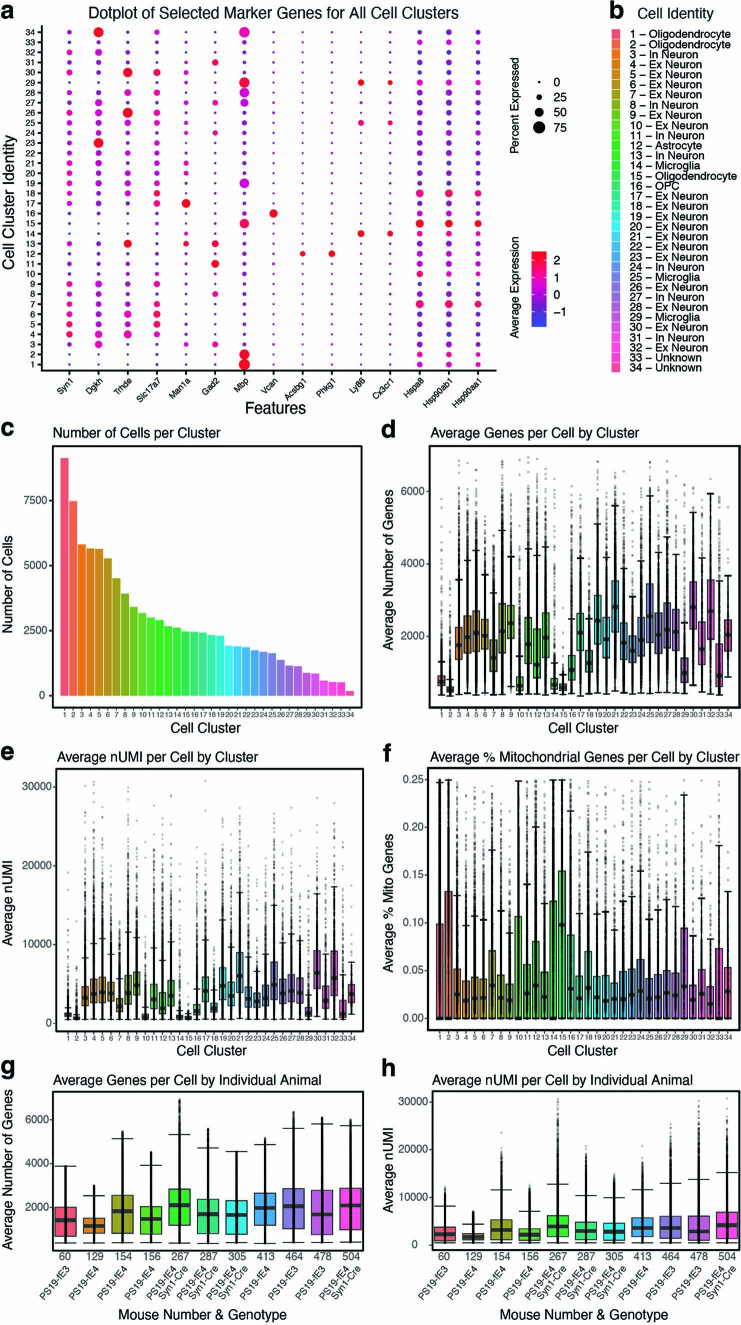
Quality control measures in snRNA-seq analysis of PS19-fE mice. **a**, Dot-plot showing the normalized average expression of selected marker genes for all 34 distinct hippocampal cell clusters. **b**, Cluster identity of the 34 identified cell types. **c**, The number of cells per cluster. **d**, Boxplot of the number of genes per cell in each cluster. Each dot represents number of genes in a single cell in that cluster. **e**, Boxplot of nUMI per cell in each cluster. Each dot represents nUMI in a single cell in that cluster. **f**, Boxplot of the percentage of mitochondrial genes per cell in each cluster. Each dot represents the percentage of mitochondrial genes in a single cell in that cluster. **g**, Boxplot of the number of genes per cell in each individual animal. Each dot represents number of genes in a single cell from that animal. **h**, Boxplot of nUMI per cell in each individual animal. Each dot represents nUMI in a single cell from that animal. Number of cells (n) for each cell cluster can be found in c. For data in d-h, the lower, middle and upper hinges of the boxplots correspond to the 25th, 50th and 75th percentiles respectively. The upper whisker of the boxplot extends from the upper hinge to the largest value no further than 1.5 * IQR from the upper hinge (where IQR is the interquartile ranger, or distance between 25th and 75th percentiles). The lower whisker extends from the lower hinge to the smallest value at most 1.5 * IQR from the lower hinge. Data beyond the end of the whiskers are outlier points. For details of the analyses, see Methods.

To gain an in-depth understanding of the cell type-specific effects of neuronal APOE4 at the transcriptomic level across different types of hippocampal cells, we performed snRNA-seq on isolated hippocampi from 10-month-old PS19-fE4 mice with or without Syn1-Cre and PS19-fE3 mice. The snRNA-seq dataset contained 95,156 nuclei covering 25,890 genes after normalization and filtering for quality control (Extended Data Fig. 4a–h). Clustering by the Louvain algorithm^58^ and visualization by Uniform Manifold Approximation and Projection (UMAP) revealed 34 distinct cell clusters (Fig. 5a). Based on their expression of marker genes, these clusters were assigned to 17 excitatory neuron (Ex) clusters (4–7, 9, 10, 17–23, 26, 28, 30 and 32), 7 inhibitory neuron (In) clusters (3, 8, 11, 13, 24, 27 and 31), three oligodendrocyte clusters (1, 2 and 15), one astrocyte cluster (12), three microglia clusters (14, 25 and 29), one OPC cluster (16) and two unknown clusters (33 and 34) (Fig. 5a, Extended Data Fig. 4a,b and Supplementary Table 1). As predicted, APOE was highly expressed in astrocytes (cluster 12) in PS19-fE4, PS19-fE3 and PS19-fE4/Syn1-Cre mice, validating that neuronal APOE4 removal by Syn1-Cre does not alter astrocytic APOE4 expression (Fig. 5b). As we reported previously^27^, some neurons also expressed APOE in PS19-fE4 and PS19-fE3 mice and neuronal APOE expression was eliminated in PS19-fE4/Syn1-Cre mice (Fig. 5b). Notably, there was a reduction in APOE expression in oligodendrocyte clusters 1, 2 and 15 and OPC cluster 16 in PS19-fE4/Syn1-Cre mice (Fig. 5b). As oligodendrocytes and OPCs did not have any evident Cre recombinase expression by immunohistochemical staining (Extended Data Fig. 1c,d) and Syn1 was highly expressed within all neuronal clusters but essentially absent from these oligodendrocyte cell clusters (Extended Data Fig. 4a), the potential reduction in APOE expression in oligodendrocytes and OPCs in PS19-fE4/Syn1-Cre mice is likely due to a secondary effect in response to the removal of neuronal APOE, which warrants further investigation in future studies.

**Extended Data Fig. 5:**
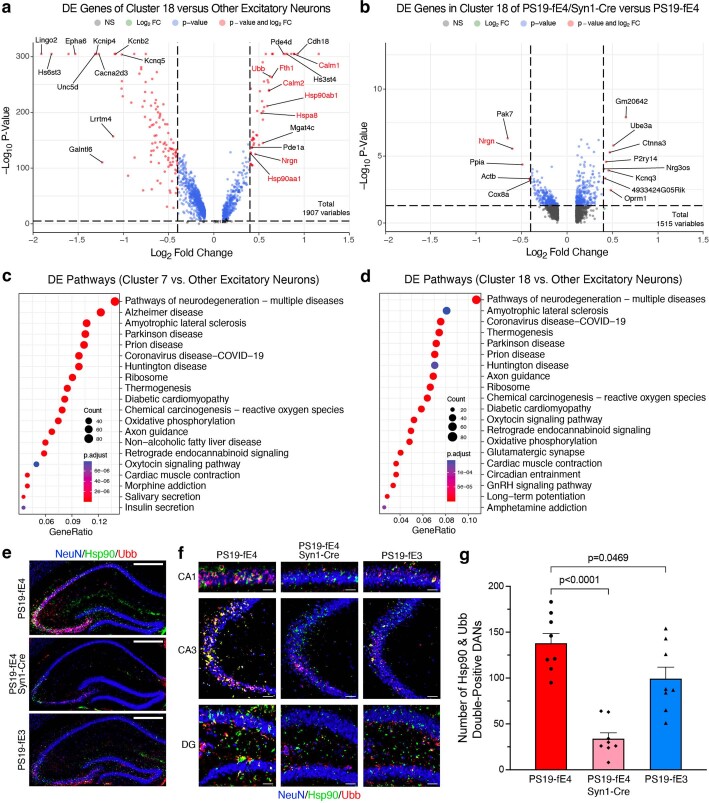
Neuronal APOE4 removal reduces disease-associated neurons (DANs). **a**, Volcano plot of the differentially expressed (DE) genes between excitatory neuron cluster 18 and all other excitatory neuron clusters. The dashed lines represent log2 fold change threshold of 0.4 and p-value threshold of 0.00001. Gene names highlighted in red text indicate they are selected marker genes for DANs. **b**, Volcano plot of the DE genes in excitatory neuron cluster 18 in PS19-fE4/Syn1-Cre mice versus PS19-fE4 mice. The dashed lines represent log2 fold change threshold of 0.4 and p-value threshold of 0.05. In a,b, the unadjusted p-values and log2 fold change values used here were generated from the gene-set enrichment analysis (see Methods for details) using the two-sided Wilcoxon Rank-Sum test as implemented in the FindMarkers function of the Seurat package. **c**,**d**, Dot-plots of the top 20 KEGG pathways significantly enriched for the DE genes of excitatory neuron cluster 7 vs other excitatory neuron clusters (c), and excitatory neuron cluster 18 vs other excitatory neuron clusters (d) using over-representation or enrichment analysis. For c,d, p-values are based on a hypergeometric test and are adjusted for multiple testing using the Benjamini-Hochberg method. The size of the dots is proportional to the number of genes in the given gene set. Gene ratio represents the proportion of genes in the respective gene set that are deemed to be differentially expressed using the FindMarkers function in Seurat. **e**, Immunohistochemical staining for NeuN and two marker proteins (Hsp90 and Ubb) that are upregulated in the disease-associated neuron clusters 7 and 18 in the hippocampus of 10-month-old PS19-fE4, PS19-fE4/Syn1-Cre, and PS19-fE3 mice (scale bar, 500 µm). **f**, High magnification images of three hippocampal subregions for staining described in (e) (scale bars, CA1 50 µm, CA3 150 µm, DG 100 µm). **g**, Quantification of the number of NeuN+ neurons that are double-positive for Hsp90 and Ubb in the hippocampus of 10-month-old PS19-fE4, PS19-fE4/Syn1-Cre, and PS19-fE3 mice. Data in g are n = 8 mice per genotype and represented as mean ± SEM, one-way ANOVA with Tukey’s post hoc multiple comparisons test. DG, dentate gyrus. Source data

Log odds ratio estimates from a generalized linear mixed-effects model to assess association with animal models (GLMM_AM) was used to identify cell clusters that were altered in PS19-fE3 and PS19-fE4/Syn1-Cre mice versus PS19-fE4 mice. This analysis revealed that excitatory neuron clusters 7 and 18 had significantly lower odds and clusters 4 and 9 had significantly higher odds of having cells from PS19-fE4/Syn1-Cre mice than from PS19-fE4 mice (Fig. 5c,d and Supplementary Table 1). Notably, clusters 7 and 18 highly expressed APOE in PS19-fE4 mice (Fig. 5b). Based on differentially expressed (DE) gene analyses, cells in neuron clusters 7 and 18 had significantly upregulated expression of the following genes relative to the other excitatory neuron clusters: three major heat shock proteins (*Hspa8*, *Hsp90aa1* and *Hsp90ab1*), calmodulin (*Calm1* and *Calm2*), calmodulin-binding protein neurogranin (*Nrgn*) and ubiquitin B (*Ubb*) (Fig. 5e,f, Extended Data Fig. 5a and Supplementary Table 1). Notably, comparison of DE genes in cluster 7 between PS19-fE4/Syn1-Cre and PS19-fE4 mice showed that removal of neuronal APOE4 led to a drastic downregulation of these top upregulated genes (Fig. 5g). Neuron cluster 18 also had significantly upregulated expression of these same set of genes and removal of neuronal APOE4 also reduced their expression, especially *Nrgn* (Extended Data Fig. 5a,b and Supplementary Table 1). This indicates that excitatory neuron clusters 7 and 18 are not only diminished in PS19-fE4/Syn1-Cre mice, but also that the removal of neuronal APOE4 led to a dramatic reversal of the expression of many top upregulated genes in these clusters. DE pathway analysis revealed the enrichment of Kyoto Encyclopedia of Gene and Genomes (KEGG) pathways related to general neurodegeneration, AD and other neurodegenerative diseases (Extended Data Fig. 5c,d and Supplementary Table 1), indicating that clusters 7 and 18 represent neuronal APOE4-promoted disease-associated neurons (nE4-DANs).

For the cohort of mice used for snRNA-seq analysis, we utilized the left hemisphere of their brains for single-nuclei isolation as well as sequencing analysis and performed extensive pathological characterizations of the right hemisphere for each mouse (Figs. 1–3). Therefore, we can assess the relationships between the transcriptomic and pathological data for each cell cluster in this cohort of mice (Fig. 5h and Supplementary Table 2). Log odds ratio estimates from another GLMM to assess associations with histopathology (GLMM_histopathology) revealed that the proportion of cells in excitatory neuron clusters 7 and 18 exhibited significant negative associations with hippocampal volume and positive associations with the coverage area of p-tau (Fig. 5h and Supplementary Table 2). Furthermore, the proportion of cells in neuronal clusters 4 and 9 that were enriched in PS19-fE4/Syn1-Cre versus PS19-fE4 mice (Fig. 5d), exhibited significant positive associations with hippocampal volume and negative associations with p-tau coverage area (Fig. 5h and Supplementary Table 2). All these associations further support the notion that neuronal clusters 7 and 18 are nE4-DANs and that neuronal clusters 4 and 9 are associated with protection against tau pathology and hippocampal degeneration. Immunostaining for NeuN^+^ neurons that were double-positive for two distinct markers of nE4-DAN clusters 7 and 18, Hsp90 and Ubb (Fig. 5e), in the hippocampus of 10-month-old mice showed that these nE4-DANs were highly present in PS19-fE4 mice and significantly decreased in PS19-fE4/Syn1-Cre and PS19-fE3 mice (Extended Data Fig. 5e–g). Of note, it has been reported that a hallmark of the neurofibrillary tangle-bearing neurons in human brains is the upregulation of Hsp90 expression^59^. Taken together, all these data illustrate that neuronal APOE4 removal diminishes the presence of the nE4-DANs and enriches the disease-protective neuronal clusters.

**Extended Data Fig. 6:**
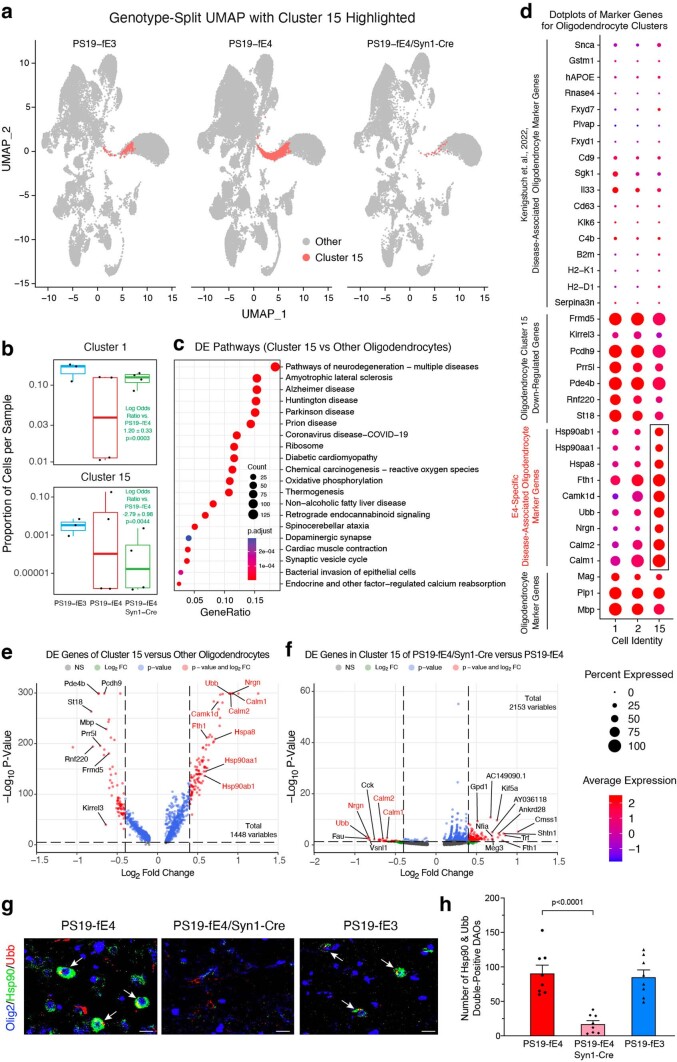
Neuronal APOE4 removal reduces disease-associated oligodendrocytes (DAOs). **a**, UMAP plot highlighting cells in hippocampal cell cluster 15. **b**, Boxplot of the proportion of cells from each sample in clusters 1 and 15 of PS19-fE4 (n = 4), PS19-fE4/Syn1-Cre (n = 4), and PS19-fE3 mice (n = 3). The lower, middle, and upper hinges of the boxplots correspond to the 25^th^, 50^th^ and 75^th^ percentiles, respectively. The upper and lower whiskers extend to the largest and smallest value no further than 1.5 * IQR, respectively. The log odds ratios are the mean ± SEM estimates of log odds ratio for oligodendrocyte clusters 1 and 15, which represents the change in the log odds of cells per sample from PS19-fE3 or PS19-fE4/Syn1-Cre mice compared to the log odds of cells per sample from PS19-fE4 mice (Supplementary Table 1). P-values are unadjusted from fits to a GLMM_AM. **c**, Dot-plots of the top 20 KEGG pathways significantly enriched for the differentially expressed (DE) genes of oligodendrocyte cluster 15 versus all other oligodendrocyte clusters (over-representation or enrichment analysis). The p-values are based on a hypergeometric test and are adjusted for multiple testing using the Benjamini-Hochberg method. **d**, Dot-plot of normalized average expression of marker genes for oligodendrocyte clusters, highlighting marker genes (boxed) of DAO cluster 15. **e**, Volcano plot of the DE genes between oligodendrocyte cluster 15 and all other oligodendrocyte clusters. Dashed lines represent log2 fold change threshold of 0.4 and p-value threshold of 0.00001. Gene names in red are marker genes for DAOs. **f**, Volcano plot of the DE genes in oligodendrocyte cluster 15 in PS19-fE4/Syn1-Cre mice versus PS19-fE4 mice. Dashed lines represent log2 fold change threshold of 0.4 and p-value threshold of 0.05. **g**, Immunohistochemical staining for Olig2 and two markers (Hsp90 and Ubb) of the DAO cluster 15 (scale bar, 50 µm). **h**, Quantification of Olig2+ oligodendrocytes double-positive for Hsp90 and Ubb. In e,f, the unadjusted p-values and log2 fold change values were generated from the gene-set enrichment analysis using the two-sided Wilcoxon Rank-Sum test. Data in g,h are from 10-month-old mice (n = 8 mice/genotype) and represented as mean ± SEM, one-way ANOVA with Tukey’s post hoc multiple comparisons test. Source data

Additionally, oligodendrocyte cluster 15 had significantly lower odds and oligodendrocyte cluster 1 had significantly higher odds of having cells from PS19-fE4/Syn1-Cre mice than from PS19-fE4 mice (Extended Data Fig. 6a,b and Supplementary Table 1). Notably, oligodendrocyte cluster 15 highly expressed APOE in PS19-fE4 mice (Fig. 5b). DE pathway analysis of cluster 15 revealed the enrichment of KEGG pathways related to general neurodegeneration, AD and other neurodegenerative diseases (Extended Data Fig. 6c and Supplementary Table 1), indicating that cluster 15 represents neuronal APOE4-promoted disease-associated oligodendrocytes (nE4-DAOs). DE gene analysis revealed that cells in oligodendrocyte cluster 15 exhibited drastically upregulated expression of *Hspa8*, *Hsp90aa1*, *Hsp90ab1*, *Calm1*, *Calm2*, *Nrgn* and *Ubb* genes (Extended Data Fig. 6d,e and Supplementary Table 1) and significantly downregulated expression of MBP (*Mbp*) and myelin-associated oligodendrocyte basic protein (*Mobp*) genes relative to the other oligodendrocyte clusters (Supplementary Table 1).

A recent study identified a subset of disease-associated oligodendrocytes (DAOs) in mouse models of neurodegenerative diseases^60^. Comparison of the marker genes of our nE4-DAOs with this previous study showed some overlap, as nE4-DAOs also had upregulation of *Snca*, *APOE*, *Fxyd7*, *B2M* and *H2-D1* gene expression compared to the other oligodendrocyte clusters (Extended Data Fig. 6d); however, we also saw dramatically upregulated (*Hspa8*, *Hsp90aa1*, *Hsp90ab1*, *Calm1*, *Calm2*, *Nrgn* and *Ubb*) and downregulated (*Frmd5*, *Kirrel3*, *Pcdh9*, *Prr5l*, *Pde4b*, *Rnf220* and *St18*) gene expression patterns that were unique to the nE4-DAOs identified in the present study, indicating that these are nE4-DAO marker genes (Extended Data Fig. 6d,e). Furthermore, comparison of DE genes in cluster 15 between PS19-fE4/Syn1-Cre and PS19-fE4 mice showed that many of the top upregulated marker genes were drastically downregulated after removal of neuronal APOE4 (Extended Data Fig. 6f). Taken together, these data indicate that neuronal APOE4 removal not only diminishes the nE4-DAO cluster 15, but also leads to a dramatic reversal of the expression of many top upregulated marker genes in nE4-DAOs.

Log odds ratio estimates from a GLMM_histopathology revealed that the proportion of cells in the nE4-DAO cluster 15 exhibited significant negative associations with hippocampal volume and MBP coverage area and positive association with the coverage area of p-tau (Fig. 5h and Supplementary Table 2). Oligodendrocyte cluster 1, which was significantly enriched in PS19-fE4/Syn1-Cre versus PS19-fE4 mice (Extended Data Fig. 6b), exhibited significant positive associations with hippocampal volume and OPC coverage area and negative associations with p-tau coverage area (Fig. 5h and Supplementary Table 2). All these associations further support the notion that oligodendrocytes in cluster 15 are nE4-DAOs and that oligodendrocytes in cluster 1 are protective oligodendrocytes against degeneration. Immunostaining for Olig2^+^ oligodendrocytes that were double-positive for two distinct markers of nE4-DAOs in cluster 15, Hsp90 and Ubb (Extended Data Fig. 6d,e), in the hippocampus of 10-month-old mice showed that these nE4-DAOs are highly present in PS19-fE4 mice and significantly decreased in PS19-fE4/Syn1-Cre mice (Extended Data Fig. 6g,h). Taken together, all these data illustrate that neuronal APOE4 removal diminishes the presence of the nE4-DAOs and enriches the disease-protective oligodendrocytes.

### Gliosis is drastically reduced after removal of neuronal APOE4

**Fig. 6:**
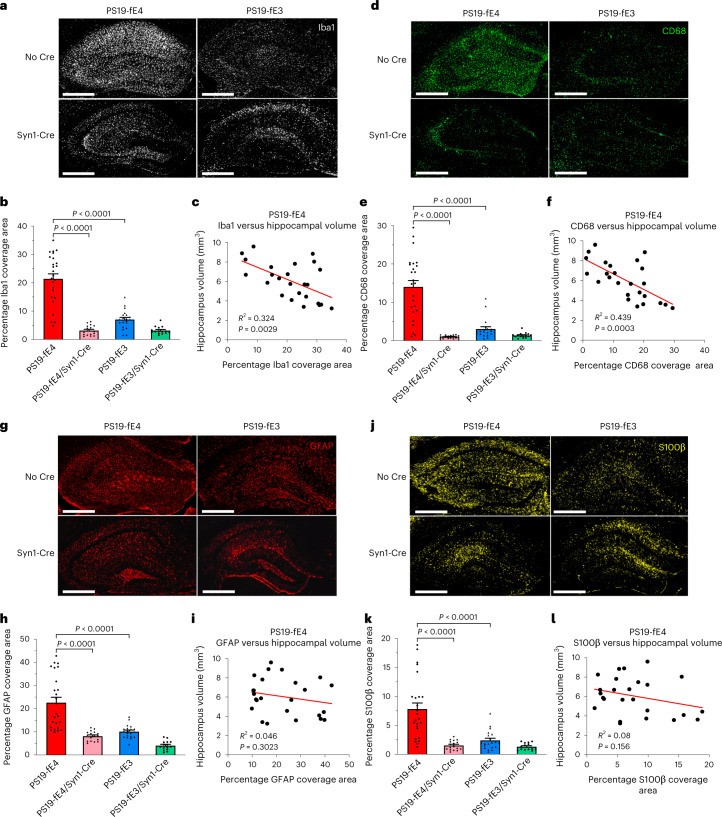
Microgliosis and astrogliosis are significantly reduced after neuronal APOE4 removal. **a**, Representative images of microglia immunostaining with anti-Iba1 in the hippocampus of 10-month-old PS19-fE4 and PS19-fE3 mice with and without Cre (scale bar, 500 µm). **b**, Quantification of the percent Iba1 coverage area in the hippocampus of 10-month-old PS19-fE4 and PS19-fE3 mice with and without Cre. **c**, Correlation between percent Iba1 coverage area and hippocampal volume of PS19-fE4 mice (*n* = 25). **d**, Representative images of activated microglia immunostaining with anti-CD68 in the hippocampus of 10-month-old PS19-fE4 and PS19-fE3 mice with and without Cre (scale bar, 500 µm). **e**, Quantification of percent CD68 coverage area in the hippocampus of 10-month-old PS19-fE4 and PS19-fE3 mice with and without Cre. **f**, Correlation between percent CD68 coverage area and hippocampal volume of PS19-fE4 mice (*n* = 25). **g**, Representative images of astrocyte immunostaining with anti-GFAP in the hippocampus of 10-month-old PS19-fE4 and PS19-fE3 mice with and without Cre (scale bar, 500 µm). **h**, Quantification of percent GFAP coverage area in the hippocampus of 10-month-old PS19-fE4 and PS19-fE3 mice with and without Cre. **i**, Correlation between percent GFAP coverage area and hippocampal volume of PS19-fE4 mice (*n* = 25). **j**, Representative images of activated astrocyte immunostaining with anti-S100β in the hippocampus of 10-month-old PS19-fE4 and PS19-fE3 mice with and without Cre (scale bar, 500 µm). **k**, Quantification of percent S100β coverage area in the hippocampus of 10-month-old PS19-fE4 and PS19-fE3 mice with and without Cre. **l**, Correlation between percent S100β coverage area and hippocampal volume of PS19-fE4 mice (*n* = 25). For quantifications in **b**,**e**,**h**,**k**, PS19-fE4: No Cre, *n* = 25; Syn1-Cre, *n* = 17; and PS19-fE3: No Cre, *n* = 20; Syn1-Cre, *n* = 15 mice. All data are represented as mean ± s.e.m., one-way ANOVA with Tukey’s post hoc multiple comparisons test. Pearson’s correlation analysis (two-sided). Source data. Source data

As it has been suggested that glial cells drive degeneration in this tauopathy model^11^, we then investigated the effect of neuronal APOE4 on gliosis. We examined the extent of microgliosis and astrogliosis within these various genotype groups at 10 months of age. PS19-fE4 mice had a significantly higher coverage area of Iba1^+^ microglia and CD68^+^ activated microglia^61^ in the hippocampus relative to PS19-fE3 mice and both were significantly reduced in PS19-fE4/Syn1-Cre mice (Fig. 6a,b,d,e). There was no significant reduction after removal of neuronal APOE3 (Fig. 6a,b,d,e). These data indicate that microgliosis is strongly increased by APOE4 relative to APOE3 in the setting of tauopathy and that removal of APOE4 from neurons attenuates the extent of microgliosis.

There was also a strong negative correlation between Iba1 coverage area and hippocampal volume in PS19-fE4 mice (Fig. 6c). Interestingly, of all pathological correlations made in PS19-fE4 mice, the coverage area of CD68^+^ activated microglia had the strongest negative correlation with hippocampal volume (Fig. 6f), suggesting that the extent of microglial activation is the strongest indicator and potential contributor to APOE4-promoted hippocampal degeneration in tauopathy.

We next assessed the extent of astrogliosis after removal of APOE from neurons. PS19-fE4 mice exhibited a significantly higher coverage area of GFAP^+^ astrocytes and S100β^+^ activated astrocytes^62^ in the hippocampus relative to PS19-fE3 mice and both were greatly reduced after removal of neuronal APOE4 (Fig. 6g,h,j,k). There was no obvious difference in the extent of astrogliosis after removal of neuronal APOE3 (Fig. 6g,h,j,k). Neither the coverage area of astrocytes nor activated astrocytes was significantly correlated with hippocampal volume in this cohort (Fig. 6i,l). These data indicate that APOE4 strongly enhances astrogliosis relative to APOE3 in tauopathy and that the removal of APOE4 from neurons eliminates this phenotype.

### Removal of neuronal APOE4 increases disease-protective and decreases disease-associated astrocytes

**Fig. 7:**
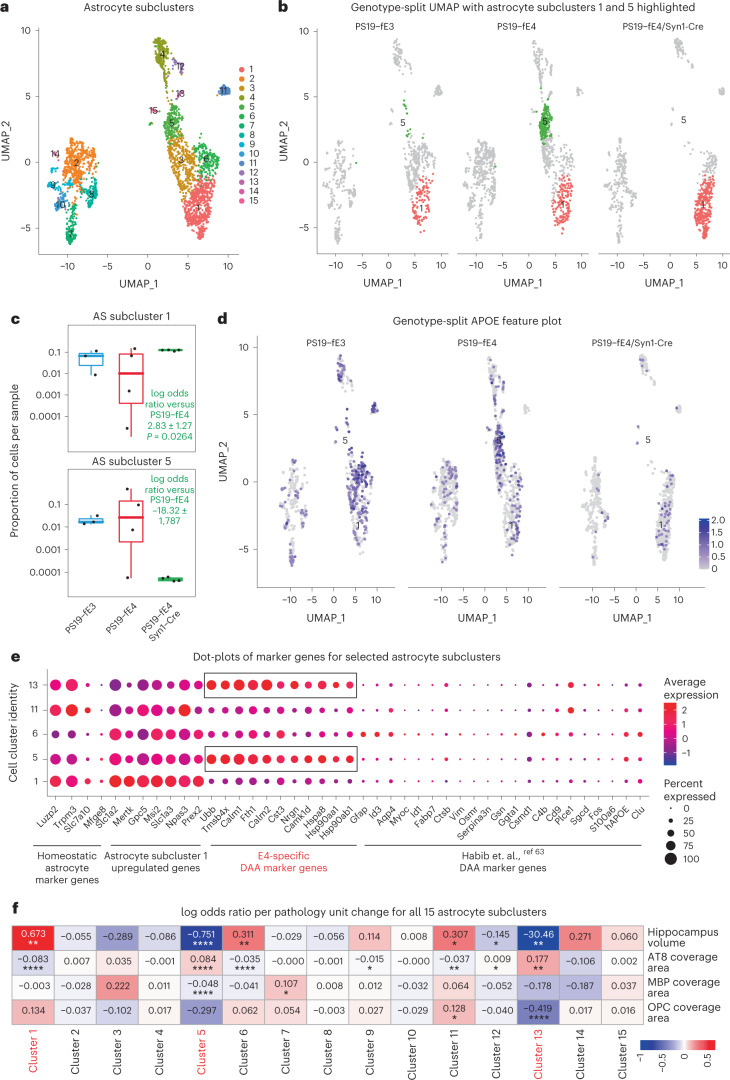
Neuronal APOE4 removal increases disease-protective astrocytes and decreases disease-associated astrocytes. **a**, UMAP plot of 15 astrocyte subclusters after subclustering hippocampal cell cluster 12. **b**, UMAP plot highlighting cells in astrocyte subclusters 1 and 5 in PS19-fE4 mice with no Cre (*n* = 4), or with Syn1-Cre (*n* = 4) and PS19-fE3 mice with no Cre (*n* = 3). **c**, Box plot of the proportion of cells from each sample in astrocyte subclusters 1 and 5. PS19-fE4 mice with no Cre (*n* = 4), or with Syn1-Cre (*n* = 4) and PS19-fE3 mice with no Cre (*n* = 3). The lower, middle and upper hinges of the box plots correspond to the 25th, 50th and 75th percentiles, respectively. The upper whisker of the boxplot extends from the upper hinge to the largest value no further than 1.5 × IQR from the upper hinge. The lower whisker extends from the lower hinge to the smallest value at most 1.5 × IQR from the lower hinge. Data beyond the end of the whiskers are outlier points. The log odds ratios are the mean ± s.e.m. estimates of log odds ratio for astrocyte subclusters 1 and 5, which represents the change in the log odds of cells per sample from PS19-fE4/Syn1-Cre mice belonging to the respective clusters compared to the log odds of cells per sample from PS19-fE4 mice. There are no cells from PS19-fE4/Syn1-Cre mice in astrocyte subcluster 5, so statistical significance for the log odds ratio is not reported (Supplementary Table 3). **d**, Feature plot illustrating the relative levels of normalized human *APOE* gene expression across all astrocyte subclusters for each mouse genotype group. **e**, Dot-plot of normalized average expression of marker genes for selected astrocyte subclusters, highlighting genes that are significantly upregulated and downregulated in astrocyte subclusters 5 and 13. **f**, Heat map plot of the log odds ratio per unit change in each pathological parameter for all astrocyte subclusters, with clusters that have significantly increased or decreased logs odds ratio after neuronal APOE4 removal highlighted in red (refer to **c**). Negative associations are shown in blue and positive associations are shown in red. Unadjusted *P* values in **c** are from fits to a GLMM_AM and unadjusted *P* values in **f** are from fits to a GLMM_histopathology (Supplementary Table 4 and Methods provide further details); the associated tests implemented in these model fits are two-sided. All error bars represent the s.e.m. AS, astrocytes.

**Extended Data Fig. 7:**
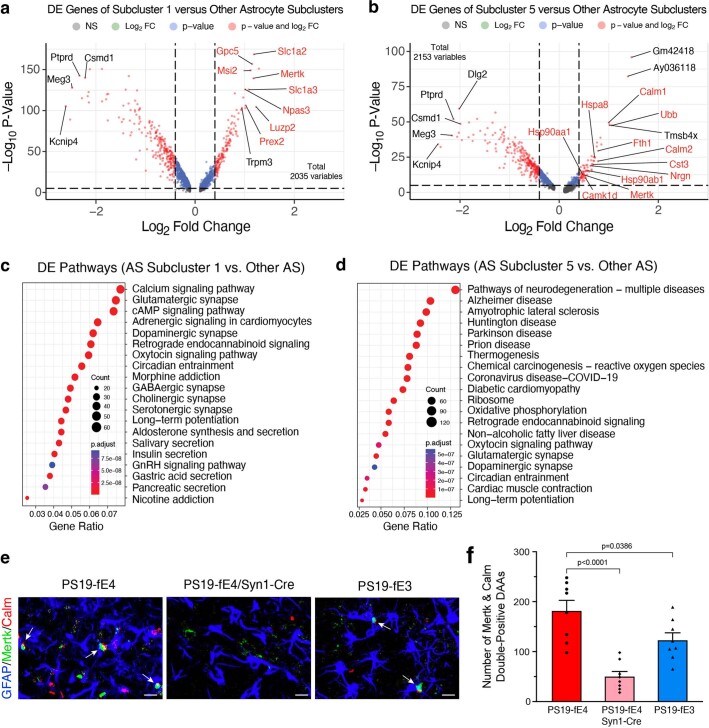
Neuronal APOE4 removal reduces disease-associated astocytes (DAAs). **a**, Volcano plot of the differentially expressed (DE) genes between astrocyte subcluster 1 and all other astrocyte subclusters. Dashed lines represent log2 fold change threshold of 0.4 and p-value threshold of 0.00001. Gene names highlighted in red text indicate they are selected marker genes for DAAs. **b**, Volcano plots of the DE genes between astrocyte subcluster 5 and all other astrocyte subclusters. Dashed lines represent log2 fold change threshold of 0.4 and p-value threshold of 0.00001. **c**, Dot-plot of the top 20 KEGG pathways significantly enriched for the DE genes of astrocyte subcluster 1 versus all other astrocyte clusters (using over-representation or enrichment analysis). **d**, Dot-plot of the top 20 KEGG pathways significantly enriched for the DE genes of astrocyte subcluster 5 versus all other astrocyte subclusters (using over-representation or enrichment analysis). **e**, Immunohistochemical staining for GFAP and two markers (Mertk and Calm) that are upregulated in the disease-associated astrocyte subcluster 5 in the hippocampus of 10-month-old PS19-fE4, PS19-fE4/Syn1-Cre, and PS19-fE3 mice (scale bar, 50 µm). **f**, Quantification of the number of GFAP + astrocytes that are double-positive for Mertk and Calm in the hippocampus of 10-month-old PS19-fE4, PS19-fE4/Syn1-Cre, and PS19-fE3 mice. For data in a,b, the unadjusted p-values and log2 fold change values used here were generated from the gene-set enrichment analysis (see Methods for details) using the two-sided Wilcoxon Rank-Sum test as implemented in the FindMarkers function of the Seurat package. For data in c,d, the p-values are based on a hypergeometric test and are adjusted for multiple testing using the Benjamini-Hochberg method. The size of the dots is proportional to the number of genes in the given gene set. Gene ratio represents the proportion of genes in the respective gene set that are differentially expressed using the FindMarkers function in Seurat. Data in f are n = 8 mice per genotype and represented as mean ± SEM, one-way ANOVA with Tukey’s post hoc multiple comparisons test. Source data

To gain deeper insights into the effects of neuronal APOE4 on subtypes of glial cells, we did further subclustering analyses of our snRNA-seq dataset. Subclustering of astrocytes (cluster 12 in Fig. 5a) identified 15 astrocyte subpopulations (Fig. 7a). Log odds ratio estimates from a GLMM_AM revealed that astrocyte subcluster 1 had significantly higher odds, whereas subcluster 5 had significantly lower odds of having cells from PS19-fE4/Syn1-Cre mice than from PS19-fE4 mice, with a complete elimination of subcluster 5 in PS19-fE4/Syn1-Cre mice (Fig. 7b,c and Supplementary Table 3). Notably, astrocyte subcluster 5 highly expressed APOE (Fig. 7d). DE gene analysis revealed that astrocyte subcluster 1 highly expressed the homeostatic astrocyte genes *Luzp2*, *Trpm2*, *Slc7a10* and *Gpc5*, while astrocyte subcluster 5 had downregulated expression of these homeostatic genes and drastic upregulated gene expression of *Hsp8a*, *Hsp90aa1*, *Hsp90ab1*, *Calm1*, *Calm2*, *Nrgn* and *Ubb* relative to other astrocyte subclusters (Fig. 7e, Extended Data Fig. 7a,b and Supplementary Table 3). As astrocyte subcluster 5 was completely eliminated in PS19-fE4/Syn1-Cre mice (Fig. 7b), we could not analyze the effect of removing neuronal APOE4 on the marker gene expression in this subcluster.

DE pathway analysis (Supplementary Table 3) revealed the enrichment of KEGG pathways related to calcium and cAMP signaling, synaptic function and long-term potentiation in astrocyte subcluster 1 (Extended Data Fig. 7c), suggesting that this subcluster is associated with supporting synaptic function. Conversely, astrocyte subcluster 5 showed an enrichment of KEGG pathways related to general neurodegeneration, AD and other neurodegenerative diseases (Extended Data Fig. 7d), indicating that this subcluster represents neuronal APOE4-promoted disease-associated astrocytes (nE4-DAAs). Comparison with a recent study that described a subset of DAAs in mouse models of AD showed that nE4-DAAs exhibit a similar upregulation of several specific marker genes, such as *Ctsb*, *Vim* and *APOE*, as observed in the previously described subset of DAAs^63^. Still, we saw a strong upregulation of a set of marker genes (*Hspa8*, *Hsp90aa1*, *Hsp90ab1*, *Calm1*, *Calm2*, *Nrgn* and *Ubb*) unique to the nE4-DAAs identified in this study (Fig. 7e), suggesting that these are nE4-DAA marker genes.

Log odds ratio estimates from a GLMM_histopathology revealed that the proportion of cells in astrocyte subcluster 5 exhibited significant negative associations with hippocampal volume and MBP coverage area and a significant positive association with the coverage area of p-tau (Fig. 7f and Supplementary Table 4), suggesting that astrocyte subcluster 5 represents nE4-DAAs. Meanwhile, astrocyte subcluster 1 exhibited a significant positive association with hippocampal volume and a significant negative association with the coverage area of p-tau (Fig. 7f and Supplementary Table 4), suggesting that astrocyte subcluster 1 represents disease-protective astrocytes. Astrocyte subcluster 13 also showed very strong negative associations with hippocampal volume and OPC coverage area and positive association with p-tau coverage area (Fig. 7f). Although this cluster was completely eliminated in PS19-fE4/Syn1-Cre mice (Fig. 7a,b), it also had a very small number of cells in PS19-fE4 mice (Fig. 7a,b), making it difficult to draw a clear conclusion on the importance of this astrocyte cluster. Immunostaining for GFAP^+^ astrocytes that were double-positive for two distinct markers of nE4-DAAs in subcluster 5, Mertk and Calm (Fig. 7e and Extended Data Fig. 7b; Mertk was used to differentiate subcluster 5 from 13), in the hippocampus of 10-month-old mice showed that these nE4-DAAs were highly present in PS19-fE4 mice and were significantly reduced after removal of neuronal APOE4 in PS19-fE4/Syn1-Cre mice and, to a lesser extent, in PS19-fE3 mice (Extended Data Fig. 7e,f). Taken together, all these data illustrate that neuronal APOE4 removal diminishes the presence of the nE4-DAAs and enriches the disease-protective astrocytes.

### Removal of neuronal APOE4 increases disease-protective microglia and decreases disease-associated microglia

**Fig. 8:**
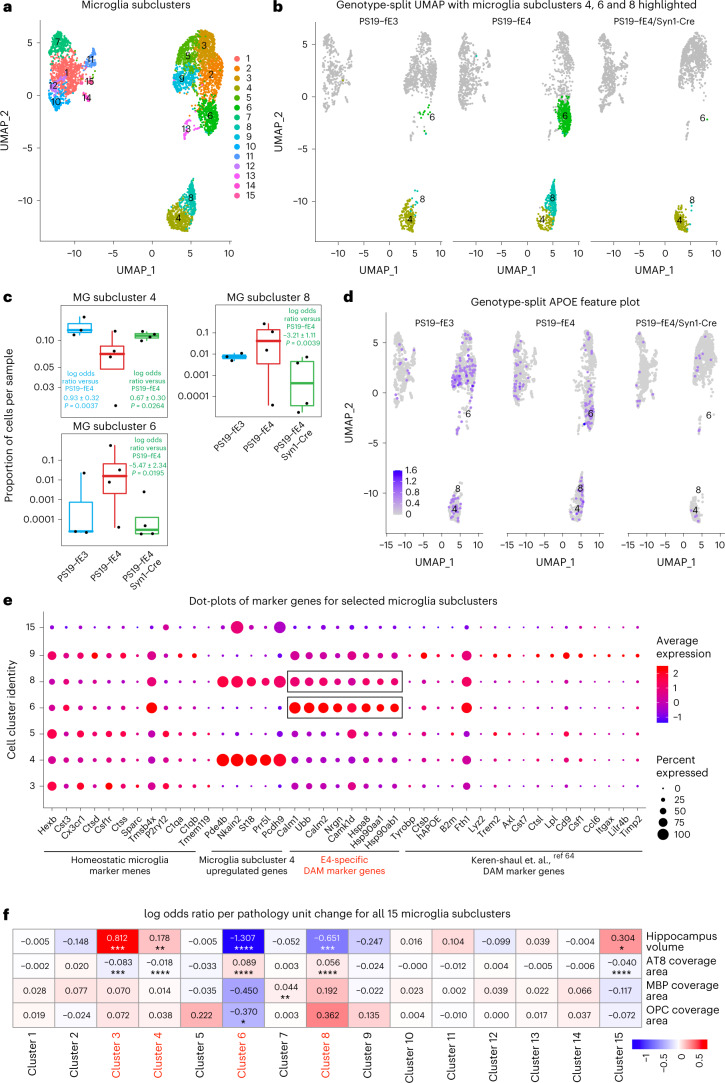
Neuronal APOE4 removal increases disease-protective microglia and decreases disease-associated microglia. **a**, UMAP plot of 15 microglia subclusters after subclustering hippocampal cell clusters 14, 25 and 29. **b**, UMAP plot highlighting cells in microglia subclusters 4, 6 and 8 in PS19-fE4 mice with no Cre (*n* = 4), Syn1-Cre (*n* = 4) and PS19-fE3 mice with no Cre (*n* = 3). **c**, Box plot of the proportion of cells from each sample in microglia subclusters 4, 6 and 8. PS19-fE4 mice with no Cre (*n* = 4), or with Syn1-Cre (*n* = 4) and PS19-fE3 mice with no Cre (*n* = 3). The lower, middle and upper hinges of the box plots correspond to the 25th, 50th and 75th percentiles, respectively. The upper whisker of the boxplot extends from the upper hinge to the largest value no further than 1.5 × IQR from the upper hinge. The lower whisker extends from the lower hinge to the smallest value at most 1.5 × IQR from the lower hinge. Data beyond the end of the whiskers are outlier points. The log odds ratios are the mean ± s.e.m. estimates of log odds ratio for microglia subclusters 4, 6 and 8, which represents the change in the log odds of cells per sample from PS19-fE3 or PS19-fE4/Syn1-Cre mice belonging to the respective clusters compared to the log odds of cells per sample from PS19-fE4 mice (Supplementary Table 5). **d**, Feature plot illustrating the relative levels of normalized human *APOE* gene expression across all microglia subclusters for each mouse genotype group. **e**, Dot-plot of normalized average expression of marker genes for selected microglia subclusters, highlighting genes that are significantly upregulated and downregulated in microglia subclusters 6 and 8. **f**, Heat map plot of the log odds ratio per unit change in each pathological parameter for all microglia subclusters, with clusters that have significantly increased or decreased logs odds ratio after neuronal APOE4 removal highlighted in red (refer to **c**). Negative associations are shown in blue and positive associations are shown in red. Unadjusted *P* values in **c** are from fits to a GLMM_AM and unadjusted *P* values in **f** are from fits to a GLMM_histopathology (Supplementary Table 6 and Methods provide details); the associated tests implemented in these model fits are two-sided. All error bars represent the s.e.m. MG, microglia.

**Extended Data Fig. 8:**
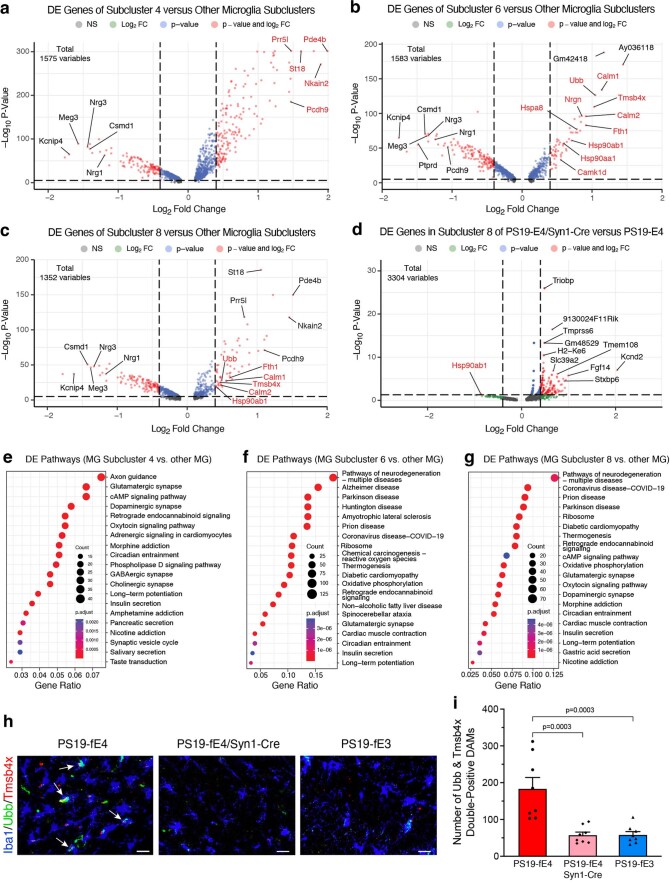
Neuronal APOE4 removal reduces disease-associated microglia (DAMs). **a**, Volcano plot of the differentially expressed (DE) genes between microglia subcluster 4 and all other microglia subclusters. Gene names in red are marker genes for DAM. Dashed lines represent log2 fold change threshold of 0.4 and p-value threshold of 0.00001. **b**, Volcano plot of the DE genes between microglia subcluster 6 and all other microglia subclusters. Dashed lines represent log2 fold change threshold of 0.4 and p-value threshold of 0.00001. **c**, Volcano plot of the DE genes between microglia subcluster 8 and all other microglia subclusters. Dashed lines represent log2 fold change threshold of 0.4 and p-value threshold of 0.00001. **d**, Volcano plot of the DE genes in microglia subcluster 8 in PS19-fE4/Syn1-Cre mice versus PS19-fE4 mice. Dashed lines represent log2 fold change threshold of 0.4 and p-value threshold of 0.05. **e**, Dot-plot of the top 20 KEGG pathways significantly enriched for the DE genes of microglia subcluster 4 versus all other microglia subclusters (over-representation or enrichment analysis). **f**, Dot-plot of the top 20 KEGG pathways significantly enriched for the DE genes of microglia subcluster 6 versus all other microglia subclusters (over-representation or enrichment analysis). **g**, Dot-plot of the top 20 KEGG pathways significantly enriched for the DE genes of microglia subcluster 8 versus all other microglia subclusters (over-representation or enrichment analysis). **h**, Immunohistochemical staining for Iba1 and two markers (Tmsb4x and Ubb) of the disease-associated microglia subclusters 6 and 8 in the hippocampus (scale bar, 50 µm). **i**, Quantification of the number of Iba1+ microglia double-positive for Tmsb4x and Ubb in the hippocampus. Mice in h,i are 10-month-old. For data in a,b,c,d, the unadjusted p-values and log2 fold change values were generated from the gene-set enrichment analysis (see Methods for details) using the two-sided Wilcoxon Rank-Sum test. For data in e,f,g, the p-values are based on a hypergeometric test and are adjusted for multiple testing using the Benjamini-Hochberg method. The size of the dots is proportional to the number of genes in the given gene set. Data in i are n = 8 mice per genotype and represented as mean ± SEM, one-way ANOVA with Tukey’s post hoc multiple comparisons test. Source data

Further subclustering of microglia (clusters 14, 25 and 29; Fig. 5a) identified 15 microglial subpopulations (Fig. 8a). Log odds ratio estimates from a GLMM_AM revealed that microglia subclusters 6 and 8 had significantly lower odds and subcluster 4 had significantly higher odds of having cells from PS19-fE4/Syn1-Cre mice than from PS19-fE4 mice (Fig. 8b,c and Supplementary Table 5). Notably, microglia subcluster 6 highly expressed APOE (Fig. 8d). DE gene analysis revealed that microglia subcluster 4 had upregulated expression of *Pde4b*, *Nkain2*, *St18*, *Prr5l* and *Pcdh9* genes, whereas microglia subclusters 6 and 8 had upregulated expression of *Hspa8*, *Hsp90aa1*, *Hsp90ab1*, *Calm1*, *Calm2*, *Nrgn* and *Ubb* genes, relative to other microglia subclusters (Fig. 8e, Extended Data Fig. 8a–c and Supplementary Table 5). Analysis of DE genes in microglia subcluster 8 between PS19-fE4/Syn1-Cre and PS19-fE4 mice showed downregulated expression of *Calm1*, *Nrgn*, *Hsp90ab1* and *Ubb* genes (Extended Data Fig. 8d and Supplementary Table 5) in PS19-fE4/Syn1-Cre mice, indicating that the removal of neuronal APOE4 not only diminishes this subcluster, but also leads to reversal of the expression of many top upregulated marker genes in this microglia subcluster. As microglia subcluster 6 was almost completely eliminated in PS19-fE4/Syn1-Cre mice (Fig. 8b), it was not possible to analyze the effect of removing neuronal APOE4 on the marker gene expression in this subcluster.

DE pathway analysis (Supplementary Table 5) revealed the enrichment of KEGG pathways related to cAMP signaling, synaptic function and long-term potentiation in microglia subcluster 4 (Extended Data Fig. 8e) and an enrichment of KEGG pathways related to general neurodegeneration, AD and other neurodegenerative diseases in subclusters 6 and 8 (Extended Data Fig. 8f,g), suggesting that subcluster 4 represents synaptic-function-supporting microglia and subclusters 6 and 8 represent neuronal APOE4-promoted disease-associated microglia (nE4-DAMs). Comparison with a recent study that described a subset of DAMs in AD mouse models showed a similar upregulation of specific gene markers *Ctsb* and *Fth1* in our nE4-DAMs as observed in the previously described subset of DAMs (Fig. 8e)^64^. On the other hand, we also observed considerable upregulation of a set of genes (*Hspa8*, *Hsp90aa1*, *Hsp90ab1*, *Calm1*, *Calm2*, *Nrgn* and *Ubb*) unique to the nE4-DAMs identified in this study (Fig. 8e), suggesting that these are nE4-DAM marker genes.

Log odds ratio estimates from a GLMM_histopathology revealed that the proportion of cells in microglia subcluster 6 and 8 both exhibited significant negative associations with hippocampal volume and significant positive associations with the coverage area of p-tau (Fig. 8f and Supplementary Table 6), suggesting that microglia subclusters 6 and 8 represent nE4-DAMs. Meanwhile, microglia subcluster 4 exhibited a significant positive association with hippocampal volume and a negative association with the coverage area of p-tau (Fig. 8f and Supplementary Table 6), suggesting that microglia subcluster 4 represents a disease-protective subpopulation of microglia. Immunostaining for Iba1^+^ microglia that were double-positive for two distinct markers of the nE4-DAMs in subclusters 6 and 8, Ubb and Tmsb4x (Fig. 8e), in the hippocampus of 10-month-old mice showed that these nE4-DAMs were highly present in PS19-fE4 mice and were significantly reduced after removal of neuronal APOE4 in PS19-fE4/Syn1-Cre mice and in PS19-fE3 mice (Extended Data Fig. 8h,i). Therefore, all these data illustrate that neuronal APOE4 removal diminishes the presence of the nE4-DAMs and enriches the disease-protective microglia.

Taken together, subclustering analyses of astrocytes and microglia illustrate that removal of neuronal APOE4 led to a drastic reduction in the amount of nE4-DAA and nE4-DAM subpopulations that have strong positive associations with tau pathology and hippocampal degeneration, while increasing the amount of disease-protective astrocyte and microglia subpopulations that have strong negative associations with tau pathology and hippocampal degeneration. This strongly supports the conclusion that neuronal APOE4 promotes the accumulation of DAAs and microglia but diminishes disease-protective astrocytes and microglia and that its removal can effectively eliminate these detrimental effects of neuronal APOE4.

## Discussion

In the present study, we investigate the roles of neuronal APOE4 in promoting the development of prominent AD pathologies in a tauopathy mouse model. We demonstrate that the removal of neuronal APOE4 has wide-ranging beneficial effects, leading to drastic reductions (1) in the accumulation and spread of pathological tau throughout the hippocampus; (2) in neurodegeneration and hippocampal neuron loss; (3) in myelin deficits and depletion of oligodendrocytes and OPCs; (4) in neuronal network hyperexcitability; (5) in microgliosis and astrogliosis and (6) in the accumulation of neurodegenerative disease-associated cell subpopulations. These findings illustrate that neuronal APOE4 is a potent driver of these AD-related pathologies and that its removal is sufficient to attenuate these disease phenotypes. Thus, our study reveals a central role of neuronal APOE4 in the pathogenesis of APOE4-driven AD and provides new insights into potential therapeutic targets to combat APOE4-related AD, such as through the removal or reduction of neuronal APOE4.

It is well established that APOE4 has a potent effect on tau pathology, as it increases tau burden in human brains^8,9,29,30^ and promotes the accumulation of p-tau in human neurons^31–33^ and mouse models^28,34,35^. We illustrate in the current study that neuronal APOE4 plays a major role in the exacerbation of tau pathology, as its removal leads to a drastic reduction in the accumulation of p-tau and neurofibrillary tangles in the hippocampus. Furthermore, tau protein has been shown to spread between cells and connected brain regions in human patients^50,65^. We show that APOE4 strongly promotes the spread of human tau protein between anatomically connected hippocampal sides relative to APOE3 and that the removal of neuronal APOE4 leads to a significant decrease in tau spread between neurons. These findings indicate that neuronal APOE4 plays a major role in promoting the accumulation and propagation of pathological tau.

We also uncover that neuronal APOE4 removal markedly reduces myelin deficits and increases the pool of mature oligodendrocytes and OPCs in the hippocampus. While myelin pathology has not been widely studied in the context of AD, impairments in myelin and oligodendrocytes have been reported in AD brains^12,39^ and in a tauopathy mouse model^17^. APOE4 has also been shown to reduce myelination and white matter integrity in human brains^39^. Our current study provides evidence that APOE4 exerts cellular source-dependent effects on myelin deficits and oligodendrocyte/OPC depletion, with neuronal APOE4 being a strong driver of this pathological phenotype in the context of tauopathy.

Neuronal APOE4 evidently plays a primary role in tau-mediated hippocampal degeneration and neuronal cell loss, as its removal significantly reduces these phenotypes and diminishes the number of postmitotic neurons undergoing apoptosis in the hippocampus. It has been suggested that microglial activation is a driving force of neurodegeneration in the setting of tauopathy^11^ and complementary to this, we observe that the removal of neuronal APOE4 reduces the extent of microgliosis and astrogliosis in the hippocampus. It is likely that neuronal APOE4 promotes gliosis, probably via increasing major histocompatibility (MHC)-I expression as we reported recently^27^, which in turn leads to neurodegeneration and myelin deficits.

The detrimental effects of neuronal APOE4 are further exemplified by snRNA-seq analysis, which reveals that the removal of neuronal APOE4 greatly diminishes the presence of neuronal APOE4-promoted disease-associated subpopulations of neurons (nE4-DANs), oligodendrocytes (nE4-DAOs), astrocytes (nE4-DAAs) and microglia (nE4-DAMs) that are enriched in APOE4-expressing tauopathy mice. Assessment of the relationships between the transcriptomic and pathological data for each mouse reveals that the accumulation of these disease-associated cell subpopulations correlates to the severity of tau pathology, neurodegeneration and myelin deficits. These data indicate that neuronal APOE4 not only drives overt pathological changes in the loss of neurons and myelin and the accumulation of activated glial cells, but it also drives subpopulations of these cell types toward unhealthy states marked by neuronal APOE4-promoted disease-associated gene signatures. The DAOs^60,66^, DAAs^63^ and DAMs^64^ have been reported in previous studies using AD mouse models or human AD brain tissues and have provided critical information that has expanded our understanding of AD pathogenesis at a cell-type-specific level; however, all these previously identified disease-associated subpopulations of cells are not justified for APOE genotype-promoted effects. Our identified nE4-DAOs, nE4-DAAs and nE4-DAMs have specific gene expression signatures promoted by neuronal expression of APOE4. Notably, these gene expression signatures focus on heat shock proteins, calmodulin and its signaling-related proteins and ubiquitin, all of which are involved in tauopathy and AD pathogenesis^59,67,68^. Further analysis and validation of these gene signatures in future studies may provide clues to the underlying pathogenic mechanism of neuronal APOE4.

Beyond this study, it is important to consider how these findings integrate with the current knowledge of the cell type-specific effects of APOE4 to better understand its pathogenic mechanisms. As reported previously, APOE is mainly produced by astrocytes and conditions of stress or aging can induce its expression in neurons^27,43^ and microglia^69,70^. While this study focused on neuronal APOE4 effects, studies from other labs have characterized the pathogenic effects of APOE4 from these different cellular sources. It has been recently shown that the removal of APOE4 from astrocytes in a tauopathy mouse model reduces the extent of tau pathology, neurodegeneration and gliosis^41^. Considering these studies side by side, it suggests that both astrocytic and neuronal APOE4 play an important role in promoting these pathologies. Still, there are some notable differences in the potency of protective effects provided by neuronal or astrocytic APOE4 removal. For instance, removal of neuronal APOE4 leads to an ~81% reduction in tau pathology, whereas removal of astrocytic APOE4 leads to a ~30% reduction in tau pathology. This potentially suggests that neuronal APOE4 may have a stronger effect than astrocytic APOE4 in driving tau pathology, although this would need to be confirmed in a comparative study using equivalent mouse models and experimental methods. Furthermore, we observe that neuronal APOE4 also leads to considerable myelin and oligodendrocyte deficits and promotes tau spreading and hippocampal network hyperexcitability and it is currently unknown if astrocytic APOE4 also plays a role in inducing these pathologies. Therefore, these studies indicate that neuronal and astrocytic APOE4 exhibit some overlap in their pathogenic actions, while we also uncover additional pathogenic effects of neuronal APOE4 that have not yet been investigated for astrocytic APOE4. It is plausible that neuronal APOE4 plays a key role in promoting the initiation of tau-induced AD pathologies and astrocytic APOE4 is important for glial response to neuronal APOE4-triggered neuron alterations. Recently, a study on microglial APOE expression in 5XFAD mice reports that its removal from microglia does not alter Aβ plaque load or number of microglia^71^, although the effect of microglial APOE on tau pathology and neurodegeneration is still unknown and requires further investigation.

In light of the findings from this study and others, it is critical to consider the most effective therapeutic strategy for targeting APOE4-driven AD. It has been debatable whether it would be most beneficial to reduce total APOE protein levels, to specifically eliminate APOE from certain cell types, or to target the pathologies induced/promoted by APOE4. A recent study utilizing anti-sense oligonucleotides (ASOs) to decrease total APOE4 levels showed significant protection against tau pathology, neurodegeneration and neuroinflammation in a P301S/APOE4 tauopathy mouse model^72^. The results from this study are encouraging and indicate that reducing total APOE levels can effectively prevent the development of AD pathologies; however, it is not clear whether the beneficial outcomes in this study are due to the reduction of APOE4 in astrocytes, neurons or both, which warrants further investigation in future studies. Considering the possibility that depletion of total APOE4 levels may have deleterious side effects^73–77^, it would be beneficial to target the specific removal of APOE4 from a certain cell type in an effort to leave the physiological functions of APOE4 largely intact while targeting its pathogenic effects. Although both neuronal and astrocytic APOE4 exert pathogenic effects, our study has shown that neuronal APOE4 has a potent effect on promoting tau pathology and more wide-ranging detrimental effects that have not yet been characterized for astrocytic APOE4, such as promoting tau spreading, myelin and oligodendrocyte deficits and network hyperexcitability. This indicates that targeted removal of neuronal APOE4 may reduce not only tau pathology and neurodegeneration, but also neuronal dysfunction and myelin deficits.

There are several options for developing cell type-specific therapies of lowering APOE4, such as using cell type-specific AAV-CRISPR-mediated gene therapy or ligand-conjugated ASOs, but further work is required in their development and efficacy assessment. Another promising therapeutic option would be to target pathologies that are induced/promoted by APOE4. It has been recently shown that microglia are strong drivers of APOE4-promoted neurodegeneration, as the depletion of microglia using PLX3397 reduces tau pathology and neurodegeneration^11^. Our study illustrates that neuronal APOE4 increases microgliosis and a previous study shows that astrocytic APOE4 also increases microgliosis^41^. Thus, the pathogenic effects of both neuronal and astrocytic APOE4 might overlap or act in concert to promote microgliosis and blocking APOE4’s downstream effects on microgliosis may be a viable therapeutic option. Still, additional studies are required to determine the best approach for targeting APOE4-induced microgliosis, as oral PLX administration has also been shown to have off-target effects that deplete mature monocytes in the bone marrow^78^, which may be beneficial or detrimental depending on the disease context.

All in all, our study identifies neuronal APOE4 as a strong driver of many important AD pathologies and demonstrates that it has a potent effect on promoting tau pathology, gliosis and accumulation of disease-associated glial subpopulations and subsequent degenerative phenotypes. Thus, neuronal APOE4 plays a central role in the pathogenesis of APOE4-related AD and should be considered as a therapeutic target for developing drugs combating APOE4’s detrimental effects in AD and other tauopathies.

## Methods

### Mice

Human LoxP-floxed APOE knock-in (fE) mice with conditional deletion of the human *APOE* gene were generated as previously described^40^. Briefly, homozygous fE3 and fE4 mice^45^ were crossbred with Synapsin-1-Cre transgenic mice (B6.Cg-Tg(Syn1-Cre)671Jxm/J) (The Jackson Laboratory, 003966)^46^. The fE/Cre mice were crossbred with tau-P301S (PS19) transgenic mice (B6;C3-Tg(Prnp-MAPT*P301S)PS19Vle/J) (The Jackson Laboratory, 008169) that express human P301S 1N4R tau driven by the PrP promoter to generate PS19-fE4 and PS19-fE3 mice with no Cre or Syn1-Cre. Littermates that were negative for Syn1-Cre were used as PS19-fE controls. For generation of the PS19-fE/Syn1-Cre line, only female Syn1-Cre mice were used for breeding purposes because germline recombination has been reported to occur in the progeny of male Syn1-Cre mice^79^. All mice were on a pure C57BL/6 genetic background and were housed in a pathogen-free barrier facility on a 12-h light cycle at 19–23 °C and 30–70% humidity. Animals were identified by ear punch under brief isoflurane anesthesia and genotyped by PCR of a tail clipping. All animals otherwise received no procedures except those reported in this study. For all studies, both male and female mice were used. All animal experiments were conducted in accordance with the guidelines and regulation of the National Institutes of Health, the University of California and the Gladstone Institutes under the protocol AN176773. All protocols and procedures followed the guidelines of the Laboratory Animal Resource Center at the University of California, San Francisco (UCSF) and the ethical approval of the UCSF institutional animal care and use committee.

The PS19-fE mice were analyzed at 10 months of age. Brain tissue was collected after mice received intraperitoneal injections of avertin (Henry Schein) and were transcardially perfused with 0.9% saline for 1 min. Depending on the study, brain tissue was fixed as whole brains or hemi-brains. For hemi-brains, the right hemispheres were drop-fixed for 48 h in 4% paraformaldehyde (Electron Microscopy Sciences), washed for 24 h in 1× PBS (Corning) and cryoprotected in 30% sucrose (Sigma) for 48 h at 4 °C. The fixed right hemispheres were cut into 30-µm thick coronal sections on a freeze sliding microtome (Leica) and stored in cryoprotectant solution at −20 °C (30% ethylene glycol, 30% glycerol and 40% 1× PBS). Left hemispheres were snap frozen on dry ice and stored at −80 °C.

### Immunohistochemistry

Several brain sections (~300 µm apart) were washed with 1× PBS-T (PBS + 0.1% Tween-20) (Millipore Sigma) and incubated for 5 min in boiling antigen retrieval buffer (Tris buffer, pH 7.6; TEKNOVA). Sections were then washed in PBS-T before being incubated in blocking solution (5% normal donkey serum (Jackson Labs), 0.2% Triton-X (Millipore Sigma) in 1× PBS for 1 h at room temperature. Then, sections were washed in PBS-T and incubated in Mouse-on-Mouse (MOM) Blocking Buffer (one drop MOM IgG in 4 ml PBS-T) (Vector Labs) for 1 h at room temperature. After MOM block, sections were incubated in primary antibody at 4 °C overnight after being diluted to optimal concentrations (anti-APOE 1:200 dilution (Cell Signaling); anti-calmodulin 1:100 dilution (Thermo Fisher); anti-CD68 1:100 dilution (Bio-Rad); anti-Cre recombinase 1:800 dilution (Cell Signaling); anti-cleaved caspase-3 1:100 dilution (Cell Signaling); anti-GFAP (ms) 1:800 dilution (Millipore Sigma); anti-GFAP (gt) 1:800 dilution (Novus Biological); anti-GFP 1:5,000 dilution (Thermo Fisher); anti-Hsp90 1:100 dilution (Abcam); anti-Iba1 (rbt) 1:300 dilution (Wako); anti-Iba1 (gt) 1:100 dilution (Abcam); anti-MBP 1:500 dilution (Abcam); anti-Mertk 1:100 dilution (Thermo Fisher); anti-NeuN 1:500 dilution (Millipore Sigma); anti-NG2 1:500 dilution (Abcam); anti-Olig2 (ms) 1:100 dilution (Millipore Sigma); anti-Olig2 (gt) 1:100 dilution (R&D Systems); anti-S100β 1:200 dilution (Abcam); anti-Tmsb4x 1:100 dilution (Thermo Fisher); anti-Ubb 1:100 dilution (Thermo Fisher)). After primary antibody incubation, sections were washed in PBS-T and incubated at room temperature for 1 h in secondary antibodies (donkey anti-mouse 488 1:1,000 dilution (Abcam); donkey anti-rabbit 488 1:1,000 dilution (Abcam); donkey anti-rat 488 1:1,000 dilution (Abcam); donkey anti-mouse 594 1:1,000 dilution (Abcam); donkey anti-rabbit 594 1:1,000 dilution (Abcam); donkey anti-guinea pig 594 1:1,000 dilution (Jackson Immuno); donkey anti-mouse 647 1:1,000 dilution (Abcam); donkey anti-rabbit 647 1:1,000 dilution (Abcam); and donkey anti-guinea pig 647 1:1,000 dilution (Jackson Immuno). Sections were washed in PBS-T and incubated in DAPI (1:30,000 dilution in PBS-T) (Thermo Fisher) for 8 min at room temperature. After washing with PBS-T, sections were mounted onto microscope slides (Fisher Scientific), coverslipped with ProLong Gold mounting medium (Vector Laboratories) and sealed with clear nail polish. Images were taken using an FV3000 confocal laser scanning microscope (Olympus) or Aperio VERSA slide scanning microscope (Leica) at ×10, ×20, ×40 or ×60 magnification depending on the stain. For each stain, all samples were stained at the same time to limit batch-to-batch variation and imaged at the same fluorescent intensity. For the percent coverage area quantification, an optimal threshold was established for each stain in ImageJ and all samples were quantified utilizing the established threshold for each stain. To exclude the possibility of bias, researchers were blinded to samples.

For DAB (3,3′-diaminobenzidine) staining, several brain sections (~300 µm apart) were washed in PBS-T and incubated for 5 min in boiling antigen retrieval buffer (1× PBS, 0.1 M sodium citrate and 0.1 M citric acid) (Fisher Scientific, Fluka). Next, sections were washed in PBS-T and incubated for 15 min in endogenous peroxidase buffer (1× PBS, 10% methanol (Fisher Scientific) and 3% H_2_O_2_ (Sigma)) and washed in PBS-T before being incubated in blocking solution (1× PBS-T, 5% normal donkey serum and 1% non-fat dry milk) for 1 h at room temperature. After blocking, sections were washed in PBS-T and incubated in avidin/biotin blockage (four drops of each block) (Vector Laboratories) for 15 min and then washed in PBS-T. Sections were incubated in MOM Blocking Buffer (one drop MOM IgG in 4 ml PBS-T) (Vector Labs) for 1 h at room temperature. Following MOM block, sections were washed in PBS-T and incubated in primary antibody at 4 °C overnight (anti-p-tau (AT8) 1:100 dilution (Invitrogen); and anti-HT7 1:200 dilution (Peter Davies)). After primary antibody incubation, sections were washed in PBS-T and incubated in biotinylated secondary antibody (1:200 dilution; Jackson Immuno) at room temperature for 1 h. Next, sections were washed in PBS-T and incubated in ABC buffer (Vector Laboratories) that was prepared 10 min before the incubation step. Sections were washed for in PBS-T and then Tris buffer (pH 7.6). Sections were incubated in DAB buffer (5 ml 1× PBS, two drops Buffer Stock Solution, two drops DAB and two drops H_2_O_2_) (Vector Laboratories) for precisely 2 min. Staining was halted by washing sections in Tris buffer (pH 7.6) and then in PBS-T. Sections were mounted onto microscope slides and dried at room temperature overnight. Next, mounted sections were submerged into Xylene (Fisher Scientific) and coverslipped with DPX mounting medium (Sigma-Aldrich). Images were taken using an Aperio VERSA slide scanning microscope (Leica) at ×10 magnification.

For Thio-S staining, several brain sections (300 µm apart) were mounted onto slides and the protocol was adapted from a previous study^50^. The tissue was washed with 1× PBS-T and then incubated in a solution of 0.06% Thio-S in PBS for 8 min. Then, sections were washed for 1 min in 80% ethanol and 5 min in PBS-T. Sections were then counterstained with DAPI for 8 min, washed with PBS-T and coverslipped. Thio-S staining was imaged in the 488-fluorescent channel on an Aperio VERSA slide scanning microscope (Leica) at ×10 magnification.

### Volumetric analysis

Hippocampal brain sections (seven sections per mouse, 30-µm thick, 300 µm apart) were mounted onto microscope slides (Fisher Scientific). A 0.1% Sudan Black solution was prepared by adding Sudan Black powder (Sigma) to 70% ethanol (KOPTEC) and mixing the solution using a magnetic stirrer. The solution was then centrifuged at 1,100*g* for 10 min and the collected supernatant was filtered using a 0.2-µm filter syringe (Thermo Scientific). Sections were then stained with the 0.1% Sudan Black solution for 10 min and washed in 70% ethanol and then in Milli-Q water. Sections were coverslipped with ProLong Gold mounting medium (Invitrogen) and imaged on an Aperio VERSA slide scanning microscope (Leica) at ×10 magnification. To quantify the volumes of the hippocampus and posterior lateral ventricle, we traced the areas of interest in ImageJ and used the formula: volume = (sum of area) × 0.3 mm (ref. ^28^). We took a sum of all seven brain sections per mouse, roughly between coordinates AP = −1.2 and AP = −3.4.

### Neuronal layer thickness measurements

Two brain sections (30-µm thick, 300 µm apart) underwent immunofluorescence staining as described above using the primary antibody NeuN (1:500 dilution) to visualize the neuronal cell layers of the hippocampus. Sections were imaged at ×20 magnification using an FV3000 confocal laser scanning microscope (Olympus). The thickness of the CA1 pyramidal cell layer and DG granular cell layer of the hippocampus were measured on the Fiji (ImageJ) software by drawing a straight line perpendicular to the NeuN^+^ cell layers at two points per hippocampal subfield and taking the average value for each mouse.

### Biochemical extraction of brain tissue

The hippocampus was dissected from snap frozen mouse hemi-brains after thawing on ice. The hippocampal tissue was weighed and homogenized using a Polytron immersion disperser homogenizer (Kinematica AG) in ice-cold RAB buffer (G Biosciences) at 10 µl mg^−1^ tissue, supplemented by phosphatase inhibitors (Roche) and protease inhibitors (Roche). Samples were then centrifuged using an Optima TLX ultracentrifuge (Beckman Coulter) at 50,000*g* for 20 min at 4 °C and the supernatant was collected as the RAB-soluble fraction. The pellets were resuspended in ice-cold RIPA buffer (Thermo Scientific) at 10 µl mg^−1^ tissue and centrifuged at 50,000*g* for 20 min at 4 °C. The supernatant was collected as the RIPA-soluble fraction and the pellet was stored at −80 °C for further use. All fractions were stored at −80 °C until further analyses.

### Western blot analysis

Biochemically extracted mouse hippocampal tissue lysates were loaded onto 12% Bis-Tris SDS–PAGE gels (Invitrogen) and separated by gel electrophoresis at 160 V using MOPS buffer. The separated proteins were transferred onto nitrocellulose membranes at 18 V for 60 min (Trans-Blot Turbo Transfer System, Bio-Rad). Membranes were washed for 3 × 5 min in PBS-T and then incubated in Intercept blocking buffer (LI-COR) for 1 h at room temperature to block nonspecific binding sites. After blocking, membranes were washed for 3 × 5 min in PBS-T and incubated with primary antibody overnight at 4 °C (AT8 at 1:3,000 dilution (Invitrogen) and TUJ1 at 1:10,000 dilution (BioLegend)). Membranes were washed for 3 × 5 min in PBS-T and incubated in fluorescently labeled secondary antibody (1:20,000 dilution; LI-COR) for 1 h in the dark at room temperature. Resulting bands were detected with the Odyssey CLx infrared imaging system (LI-COR) and the fluorescence intensity of the bands was quantified as a ratio of AT8:TUJ1 signal using Image Studio software.

### Sandwich ELISA

Hippocampal tissue lysates were diluted in H_2_O to the appropriate concentration and were run according to the provided manufacturer protocols (human APOE (Abcam) and human total tau (Thermo Fisher)). Sample reactions were read on a SpectraMaX M5 spectrophotometer (Molecular Devices) and protein concentrations were determined after interpolating a standard curve and adjusting for dilutions.

### Stereotaxic surgery on mice

After being anesthetized with an intraperitoneal injection of ketamine (60 mg kg^−1^) and xylazine (30 mg kg^−1^) and maintained on 0.8–1.0% isoflurane (Henry Schein), mice were secured in a stereotaxic alignment system model 940 using earbars and a tooth bar (Kopf Instruments). We removed hair using Nair and cut the scalp open using a scalpel and sterilized with 70% ethanol. Cranial sutures were visualized using 3% hydrogen peroxide. Following identification of Bregma, a unilateral stereotaxic site was drilled with a 0.5-mm microburr (Fine Science Tools) using coordinates *X* = +1.5, *Y* = −2.1 and *Z* = −2.1). Mice were injected with 2 µl of the respective virus (AAV2(Y444F)-smCBA-human_P301S_tau-WPRE, 2.10E + 13 vg ml^−1^, Virovek; and AAV2-synapsin-GFP, 1.0E + 13 vg ml^−1^, SignaGen) at a rate of 500 nl min^−1^ and allowed to diffuse for 3 min. Following surgery, mice were sutured with nylon monofilament non-absorbable 6-0 sutures (Henry Schein) and administered analgesics buprenorphine (0.0375 mg kg^−1^ intraperitoneally), ketophen (5 mg kg^−1^ subcutaneously) and saline (500 µl intraperitoneally). Mice were monitored on a heating pad until ambulatory and provided Hydrogel for hydration.

### Brain slices electrophysiological recordings and data analyses

For electrophysiological recording study, 8-month-old PS19-fE3 mice and PS19-fE4 mice with no Cre and Syn1-Cre were anesthetized with isoflurane and decapitated. The brain was rapidly removed and placed in ice-cold (2–5 °C) slicing solution (in mM): 110 choline chloride, 2.5 KCl, 26 NaHCO_3_, 10 MgCl_2_, 1.25 NaH_2_PO_4_, 0.5 CaCl_2_, 10 glucose, 3 Na pyruvate and 1 l-ascorbic acid, pH 7.4. The 350-µm thick sagittal slices were cut from both hemispheres using a vibratome (VT1200, Leica) and transferred to a 95% O_2_–CO_2_ vapor interface holding chamber (BSK5, Scientific Systems Design) containing artificial cerebrospinal fluid (ACSF) where they were allowed to recover at 34 °C for 1 h and held at room temperature (20–22 °C) afterwards. ACSF contained (in mM): 126 NaCl, 2.5 KCl, 1.5 CaCl_2_, 1.5 MgCl_2_, 26 NaHCO_3_, 1.25 NaH_2_PO_4_, 10 glucose and 1.5 l-ascorbic acid, pH 7.4.

For input/output recording studies, local fPSPs were elicited by orthodromic stimulation of Schaffer collaterals by concentric bipolar stimulating electrode (FHC) connected to a current stimulus isolator (NL800A, Digitimer North America) and placed in CA2 stratum radiatum. fPSPs were recorded with a glass borosilicate microelectrode filled with ACSF and placed in CA1 stratum radiatum. Signals were sampled and digitized by MultiClamp 700B amplifier and Digidata 1550B1 acquisition system with pClamp10 software (Molecular Devices) and analyzed using IgorPro6 software (Wavemetrics) running custom macros. fPSP slopes were analyzed as the linear fit slope values between 10% and 90% of fPSP peak. Input-output relationships were recorded as the fPSP slope values in response to increasing stimulation intensity (20–60 µA), with fPSP slope gain calculated as the linear slope of the resulting input–output curve.

### Single-nuclei preparation for 10x loading

The mouse hippocampus was dissected on ice and placed into a pre-chilled 2 ml Dounce with 1 ml of cold 1× Homogenization Buffer (1× HB) (250 mM sucrose, 25 mM KCl, 5 mM MgCl_2_, 20 mM tricine-KOH pH 7.8, 1 mM dithiothreitol, 0.5 mM sermidine, 0.15 mM sermine, 0.3% NP40, 0.2 U µl^−1^ RNase inhibitor and ~0.07 tablets per sample protease inhibitor). Dounce with ‘A’ loose pestle (~ten strokes) and then with ‘B’ tight pestle (~15 strokes). The homogenate was filtered using a 70-µm Flowmi strainer (Eppendorf) and transferred to a pre-chilled 2-ml LoBind tube (Fisher Scientific). Nuclei were pelleted by spinning for 5 min at 4 °C at 350 RCF. The supernatant was removed and the nuclei were resuspended in 400 µL 1X HB. Next, 400 µl of 50% Iodixanol solution was added to the nuclei and then slowly layered with 600 µl of 30% Iodixanol solution under the 25% mixture, then layered with 600 µl of 40% Iodixanol solution under the 30% mixture. The nuclei were then spun for 20 min at 4 °C at 3,000 g in a pre-chilled swinging bucket centrifuge. Then 200 µl of the nuclei band at the 30%–40% interface was collected and transferred to a fresh tube. Then, 800 µl of 2.5% BSA in PBS plus 0.2 U µl^−1^ of RNase inhibitor was added to the nuclei and then were spun for 10 min at 500 r.c.f. at 4 °C. The nuclei were resuspended with 2% BSA in PBS plus 0.2 U µl^−1^ RNase inhibitor to reach ~500 nuclei per µl. The nuclei were then filtered with a 40-µm Flowmi stainer. The nuclei were counted and then ~13,000 nuclei per sample were loaded onto 10x Genomics Next GEM chip G. The snRNA-seq libraries were prepared using the Chromium Next GEM Single Cell 3ʹ Library and Gel Bead kit v.3.1 (10x Genomics) according to the manufacturer’s instructions. Libraries were sequenced on an Illumina NovaSeq 6000 sequencer at the UCSF CAT Core.

### Custom reference genome

PS19/fE4, PS19-fE4/Syn1-Cre and PS19-fE3 mice were used for snRNA-seq. The *Homo sapiens* *MAPT* (NCBI reference sequence NM_001123066.4)^80^ and the *H.* *sapiens* *APOE* were genes of interest for this study. These genes are not expected to be a part of the mouse reference genome, so to quantify the reads aligning to these genes of interest, a custom mouse reference genome was made using the reference mouse genome sequence (GRCm38) from Ensembl (release 98)^81^ and the mouse gene annotation file from GENCODE (release M23)^82^, similar to those used in 10x Genomics CellRanger mouse reference package mm10 2020-A. The headers of the Ensembl reference mouse genome sequence fasta file with the chromosome names were modified to match the chromosome names in a fasta file from GENCODE. The annotation GTF file contains entries from non-polyA transcripts that overlap with the protein coding genes. These reads are flagged as multi-mapped and are not counted by the 10x Genomics CellRanger v.6.1.1 count pipeline^83^. To avoid this, the GTF file was modified to (1) remove version suffixes from transcript, gene and exon IDs to match the CellRanger reference packages, and (2) remove non-polyA transcripts. The *H.* *sapiens* *MAPT* sequence and *H.* *sapiens* *APOE* sequence were appended as separate chromosomes to the end of the mouse reference genome sequence and the corresponding gene annotations were appended to the filtered mouse reference gene annotation GTF file. The 10x Genomics CellRanger v.6.1.1 mkref pipeline was used to build the custom reference genome using the modified fasta and GTF file.

### Pre-processing and clustering of mouse snRNA-seq samples

The snRNA-seq samples included a total of 12 samples with four mice from each of the three genotype groups (PS19-fE4, PS19-fE4/Syn1-Cre and PS19-fE3). Each group of four mice had two male and two female mice. The demultiplexed fastq files for these samples were aligned to the custom mouse reference genome (custom reference genome methods provides additional descriptions) using the 10x Genomics CellRanger v.6.1.1 count pipeline^83^, as described in the CellRanger documentation. The include-introns flag for the count pipeline was set to true to count the reads mapping to intronic regions. The CellRanger count web summaries showed a ‘Low Fraction Reads in Cells’ error for one sample from the PS19-fE3 group, which had only ~40% reads assigned to cell-associated barcodes and <80% reads mapped to the genome. These metrics were much higher for the other 11 samples. Checking the experimental record indicated that this sample had issues at the nuclear isolation step and lower complementary DNA was recovered due to the use of an expired old batch of sample preparation reagents. All other samples were prepared with a new batch of sample preparation reagents. So, this one sample was excluded and only the remaining 11 samples were used for the downstream analyses with Seurat.

The filtered count matrices generated by the CellRanger count pipeline for 11 samples were processed using the R package for single-nucleus analysis Seurat v.4.0.5 (ref. ^84^). Each sample was pre-processed as a Seurat object and the top 1% of cells per sample with a high number of unique genes, cells with ≤200 unique genes and cells ≥0.25% mitochondrial genes were filtered out for each sample. The 11 samples were merged into a single Seurat object and normalization and variance stabilization was performed using sctransform^85^ with the ‘glmGamPoi’ (Bioconductor package v.1.6.0) method^86^ for initial parameter estimation.

Graph-based clustering was performed using the Seurat v.4.0.5 functions FindNeighbors and FindClusters. First, the cells were embedded in a *k*-nearest neighbor graph based on the Euclidean distance in the principal-component analysis space. The edge weights between two cells were further modified using Jaccard similarity. Next, clustering was performed using the Louvain algorithm implementation in the FindClusters Seurat function. Clustering was performed for all combinations of 10, 15 and 20 principal components (PCs) with 0.4, 0.5, 0.6, 0.7, 0.8 and 0.9 resolutions. Clustering with 15 PCs and 0.7 resolution resulted in 34 distinct biologically relevant clusters, which was used for further analyses.

### Cell type assignment

Data visualization using Seurat v.4.0.5 in the UMAP space for the 11 samples revealed no batch effects by age, sex, genotype, date of birth or nuclear isolation date. The marker genes for each cluster were identified using the FindAllMarkers Seurat function on the SCT assay data. This algorithm uses the Wilcoxon rank-sum test to iteratively identify DE genes in a cluster against all the other clusters. Marker genes were filtered to keep only positively expressed genes, detected in at least 25% of the cells in either population and with at least 0.5 log_2_ fold change. We assigned identities to cell clusters by matching the cell clusters to known cell types with the expression of canonical cell-type-specific genes, the expression of genes identified in publicly available mouse hippocampal single-cell RNA-seq datasets and the expression of each cluster’s marker genes in a publicly available resource of brain-wide in situ hybridization images, as we reported previously^27^.

### Subclustering of astrocytic and microglial snRNA-seq data

The hippocampal cell cluster 12 was annotated as the astrocyte cells and hippocampal cell clusters 14, 25 and 29 were annotated as the microglial cells. Both these cell types were further subclustered. Normalization and variance stabilization was performed using sctransform^85^ with the ‘glmGamPoi’ (Bioconductor package v.1.6.0) method^86^ for initial parameter estimation. Graph-based clustering was performed using the Seurat v.4.0.5 functions FindNeighbors and FindClusters. First, the cells were embedded in a *k*-nearest neighbor graph based on the Euclidean distance in the principal-component analysis space. The edge weights between two cells were further modified using Jaccard similarity. Next, clustering was performed using the Louvain algorithm implementation in the FindClusters Seurat function. Clustering was performed for all combinations of 10, 15, 20, 25 and 30 PCs with 0.4, 0.5, 0.6, 0.7, 0.8 and 0.9 resolutions. Subclustering with 15 PCs and 0.9 resolution resulted in 15 distinct biologically relevant subclusters for astrocytes. Subclustering with 15 PCs and 0.9 resolution resulted in 15 distinct biologically relevant microglia subclusters.

### Gene-set enrichment analysis

DE genes between clusters of interest were identified using FindMarkers Seurat function on the SCT assay data. This algorithm uses the Wilcoxon rank-sum test to identify DE genes between two populations. DE genes were limited to genes detected in at least 10% of the cells in either population and with at least 0.1 log_2_ fold change. Volcano plots with log_2_ fold change and unadjusted *P* value from the DE gene lists were generated using the EnhancedVolcano R package^87^. The R package org.Mm.eg.db v.3.14.0 has genome wide annotation for mouse and was used to map the gene symbols of the reported DE genes to Entrez gene IDs. Over-representation (or enrichment) analysis was performed using clusterProfiler v.4.2.1 (ref. ^88^) to find gene sets in the KEGG database^89^ for mouse associated with Entrez gene IDs of the DE genes. *P* values are based on a hypergeometric test and are adjusted for multiple testing using the Benjamini–Hochberg method^90^. Significantly enriched gene sets were filtered to have an adjusted *P* value <0.8 and at least ten DE genes present in the gene set. The same method was used for gene-set enrichment analysis of astrocyte subclusters and microglia subclusters.

### Association between clusters and genotype

A Generalized Linear Mixed-Effects Model to assess association with Animal Models (GLMM_AM) was implemented in the lme4 (v.1.1-27.1) R package^91^ and used to estimate the associations between cluster membership and the mouse model. These models were run separately for each cluster of cells. The GLMM was performed with the family argument set to the binomial probability distribution and with the bobyqa control optimizer used for the maximum likelihood estimation. Cluster membership for each cell was modeled as a 0–1 response variable according to whether or not the cell belongs to the cluster under consideration. The corresponding mouse id from which the cell was derived was the random effect variable and the animal model for this mouse ID was included as the fixed variable. The reference animal model was set to PS19-fE4. The resulting *P* values for the estimated log odds ratio across the two animal models (with respect to the PS19-fE4) and clusters were adjusted for multiple testing using the Benjamini–Hochberg method^90^. The same method was used for estimating the between cluster association with genotype for astrocyte subclusters and microglia subclusters. The proportion of cells from a sample in a given cluster were calculated by adding a pseudo count of 0.01 to the number of cells from a sample in a given cluster and dividing by the total number of cells from a sample. These proportion values were plotted as a box plot grouped by genotype using the R packages ggplot2 v.3.3.5, dplyr v.1.0.7, gridExtra v.2.3, magrittr v.2.0.1 and gdata v.2.18.0.

### Association between proportion of cell types and histopathological parameters

GLMM_histopathology was implemented in the lme4 (v.1.1–27.1) R package^91^ and used to identify cell types whose proportions are significantly associated with changes in histopathology across the samples. These models were performed separately for each combination of the cluster of cells and the four histological parameters: hippocampal volume (mm^3^), the percent of AT8 coverage area, the percent of MBP coverage area and the percent of OPC coverage area. The GLM model was performed with the family argument set to the binomial probability distribution family and with the ‘bobyqa’ control optimizer used for the maximum likelihood estimation. Cluster membership for each cell was modeled as a 0–1 response variable according to whether or not the cell belongs to the cluster under consideration. The corresponding mouse model from which the cell was derived was included as a random effect and further the mouse ID within the given mouse model was modeled as a random effect as well. Note, this represents the hierarchical nature of this data for the GLMM and the mouse models are first assumed to be sampled from a ‘universe’ of mouse models, this is then followed by sampling mice within each mouse model. The modeling choice of including the mouse model as a random effect as opposed to a fixed effect is meant to increase the degrees of freedom (or maximize the statistical power) to detect the association of interest, particularly in light of the relatively small number of replicates (3–4) per animal model. The histological parameter under consideration was modeled as a fixed effect in this model.

We visualized the log odds ratio estimates (derived from the GLMM fits) in a heat map (Fig. 5h) using pheatmap package v.1.0.12 after adjusting the *P* values distribution across histopathological parameters across cell types with Benjamini–Hochberg multiple testing correction^90^. We applied the pipeline to the astrocyte and microglia subtypes and visualized the associations between astrocyte and microglia subtypes and the four histopathological parameters in Figs. 7f and 8f, respectively.

### Immunohistochemical validation of neuronal APOE4-promoted disease-associated subpopulations of cells

Two hemibrain sections (30-µm thick, 300 µm apart) with hippocampus from each mouse underwent immunofluorescence staining as described above using antibodies against the appropriate cell type-specific marker and two distinct nE4-DA marker genes (nE4-DANs: NeuN (1:500 dilution), Hsp90 (1:100 dilution) and Ubb (1:100 dilution); nE4-DAOs: Olig2 (1:100 dilution), Hsp90 (1:100 dilution) and Ubb (1:100 dilution); nE4-DAAs: GFAP (1:800 dilution), Mertk (1:100 dilution) and Calm (1:100 dilution); nE4-DAMs: Iba1 (1:100 dilution), Ubb (1:100 dilution) and Tmsb4x (1:100 dilution)). We immunostained all mice used in the snRNA-seq experiment (PS19-fE4, *n* = 4; PS19-fE4/Syn1-Cre, *n* = 4; and PS19-fE3, *n* = 3) and also immunostained additional mice to have *n* = 8 total for each genotype included in this analysis. Sections were imaged at ×10 magnification on an Aperio VERSA slide scanning microscope (Leica) or ×40 or ×60 magnification using an FV3000 confocal laser scanning microscope (Olympus). Utilizing Fiji (ImageJ) software, we first generated a mask of the channel containing the cell type-specific marker. Then, the image calculator function was used to generate a new image that contains only cells that are positive for both distinct nE4-DA marker genes by selecting the images for both marker genes and using the operation ‘AND’. We then took the masked cell type-specific marker image and the combined nE4-DA marker gene image and utilized the image calculator function with the operation ‘AND’ to generate a new image that combines all three stains. Ultimately, this quantification method provided a measure of cells that were double-positive for both nE4-DA distinct marker genes and were also positive for the appropriate cell type-specific marker.

### General statistics and reproducibility

The differences between genotype groups were evaluated by ordinary one-way ANOVA with Tukey’s multiple comparisons test, where the mean of each column was compared to the mean of every other column. All plotted data are presented as the mean ± s.e.m. Data distribution was assumed to be normal but this was not formally tested. The correlations between two data in the same genotype group were analyzed using simple linear regression. The analyses were performed and plots were created with GraphPad Prism v.9.2.0.

For immunohistochemical, electrophysiological and biochemical analyses, sample sizes were determined using effect sizes estimated from pilot cohorts and/or previous studies to achieve ≥80% power to observe differences of >20% between genotype groups with a two-sided significance of 0.05. Researchers were blinded to mouse genotypes during these studies. No randomization method was used for the allocation of mice to study groups and no animals or data points were excluded from these studies.

For mouse snRNA-seq studies, sample sizes were determined by a power analysis using effect sizes estimated from our previous studies and a literature search. All mice used in the snRNA-seq study had undergone rigorous pathological characterization and we selected mice in each genotype group that represented near the quantified average for all pathological parameters for that genotype group. Due to the variability of pathology in certain genotype groups, specifically selecting mice that are good representatives of each genotype group for sequencing analysis allowed us to make more accurate correlations between pathologies and sequencing data. Nuclei were isolated from four mice per mouse genotype group to ensure an *n* of ≥3 mice per genotype, resulting in a total of 12 samples. Sample preparation was successful for 11 out of 12 samples. One sample, initially processed on a separate day, had low quality and quantity of cDNA recovery due to expired reagents and was excluded from downstream analyses with Seurat. All other 11 samples were prepared with a new batch of sample preparation reagents and had high quality and quantity of cDNA recovery. See ‘Pre-processing and clustering of mouse snRNA-seq samples’ section above for more details. Investigators were not blinded during analysis of the snRNA-seq datasets, as sample metadata were needed to conduct any comparisons.

The effects of APOE4 versus APOE3 on tau pathology, hippocampal degeneration, astrogliosis and microgliosis in the same tauopathy mouse model were replicated in a different cohort of mice. The snRNA-seq study was only performed in one cohort of mice.

### Reporting summary

Further information on research design is available in the Nature Portfolio Reporting Summary linked to this article.

## Supplementary information

Supplementary Information Table of contents and introduction of six supplementary Tables.

Reporting Summary

Supplementary Table 1 Marker genes for each of the 34 clusters and the lists of DE genes for various comparisons between clusters and genotypes.

Supplementary Table 2 Associations between proportion of cell types in 34 clusters and four histopathological parameters.

Supplementary Table 3 Lists of DE genes for various comparisons between astrocyte subclusters and genotypes.

Supplementary Table 4 Associations between proportion of cell types in 15 astrocyte subclusters and four histopathological parameters.

Supplementary Table 5 Lists of DE genes for various comparisons between microglia subclusters and genotypes.

Supplementary Table 6 Associations between proportion of cell types in 15 microglia subclusters and four histopathological parameters.

## Data Availability

The *H.* *sapiens* MAPT sequence is available at https://www.ncbi.nlm.nih.gov/nuccore/NM_001123066. The reference mouse genome sequence (GRCm38) from Ensembl (release 98) is available at http://ftp.ensembl.org/pub/release-98/fasta/mus_musculus/dna/Mus_musculus.GRCm38.dna.primary_assembly.fa.gz. The reference mouse gene annotation file from GENCODE (release M23) is available at http://ftp.ebi.ac.uk/pub/databases/gencode/Gencode_mouse/release_M23/gencode.vM23.primary_assembly.annotation.gtf.gz. The KEGG pathways database is available at https://www.genome.jp/kegg/pathway.html. The snRNA-seq datasets generated during the study are available at the Gene Expression Omnibus under accession number GSE221215. Data associated with Figs. 5, 7 and 8 and Extended Data Figs. 4–8 are also available in the Supplementary Information. Source data are provided with this paper.

## Source data

Source Data Fig. 1 Statistical source data for Fig. 1b,d,g,h,m,o.
Source Data Fig. 1 Uncropped scans of western blot gel image source data for Fig. 1e,f.
Source Data Fig. 2 Statistical source data for Fig. 2b,c,e,g,i–l.
Source Data Fig. 3 Statistical source data for Fig. 3b,d,f.
Source Data Fig. 4 Statistical source data for Fig. 4a,b.
Source Data Fig. 6 Statistical source data for Fig. 6b,c,e,f,h,i,k,l.
Source Data Extended Data Fig. 1 Statistical source data for Extended Data Fig. 1i.
Source Data Extended Data Fig. 2 Statistical source data for Extended Data Fig. 2f–j.
Source Data Extended Data Fig. 5 Statistical source data for Extended Data Fig. 5g.
Source Data Extended Data Fig. 6 Statistical source data for Extended Data Fig. 6h.
Source Data Extended Data Fig. 7 Statistical source data for Extended Data Fig. 7f.
Source Data Extended Data Fig. 8 Statistical source data for Extended Data Fig. 8i.

## Code availability

The following packages/software were used either as dependencies to downloading or using packages mentioned in the Methods section or in creating the figures in this manuscript: Seurat v.4.0.4, Seurat v.4.0.5, CellRanger v.3.0.2, CellRanger v.6.1.1, glmGamPoi v.1.6.0, clusterProfiler v.4.2.1, lme4 v.1.1-27.1, factoextra v.1.0.7, ggplot2 v.3.3.5, dplyr v.1.0.7, gridExtra v.2.3, magrittr v.2.0.1, gdata v.2.18.0, org.Mm.eg.db v.3.14.0, pheatmap v.1.0.12, EnhancedVolcano v.1.14.0, R v.4.1.0, R v.4.1.2 and Fiji v.1.0 (ImageJ). Western blot images were analyzed using Image Studio Lite v.5.2.5 (LI-COR). GraphPad Prism v.9.2.0 was used for some statistical analyses and graphing. All codes generated with custom R and shell scripting during this study are accessible via GitHub at https://github.com/ADNetworksPPG/YH_NK01_NatureAging_paper and on Zenodo at 10.5281/zenodo.7508271.
