# Supplementary material for: Neuronal APOE4 removal protects against tau-mediated gliosis, neurodegeneration and myelin deficits
Source: Nat Aging. 2023 Feb 20;3(3):275–96. doi: 10.1038/s43587-023-00368-3 (PMC10154214; doi:10.1038/s43587-023-00368-3)
Supplement: Supplementary file 1 — Table of contents and introduction of six supplementary Tables. [file 43587_2023_368_MOESM1_ESM.pdf]

nature aging

Article https://doi.org/10.1038/s43587-023-00368-3

Neuronal APOE4 removal protects against tau-mediated gliosis, neurodegeneration and myelin deficits

In the format provided by the authors and unedited

Table of Contents of Supplementary Information

Supplementary Table 1

The marker genes for each of the 34 clusters in Supplementary Table 1a are identified by the cell-type assignment analysis (see Methods for details) using the two-sided Wilcoxon Rank Sum test as implemented in the FindAllMarkers Seurat function. Supplementary Table 1d–l are the lists of differentially expressed genes for various comparisons between clusters and genotypes as identified by the gene-set enrichment analysis (see Methods for details) using the two-sided Wilcoxon Rank Sum test as implemented in the FindMarkers function of the Seurat package.

Supplementary Table 2

Associations between proportion of cell types in 34 clusters and four histopathological parameters. For each cluster, the table reports the logOddsRatio for unit change in histopathology, the corresponding standard error, unadjusted p-values, and FDR adjusted p-values after fitting a Generalized Linear Mixed-Effects Model (GLMM_histopathology).

Supplementary Table 3

Supplementary Table 3c–f are the lists of differentially expressed genes for various comparisons between astrocyte subclusters as identified by the gene-set enrichment analysis (see Methods for details) using the two-sided Wilcoxon Rank Sum test as implemented in the FindMarkers function of the Seurat package.

Supplementary Table 4

Associations between proportion of cell types in 15 astrocyte subclusters and four histopathological parameters. For each astrocyte subcluster, the table reports the logOddsRatio for unit change in histopathology, the corresponding standard error, unadjusted p-values, and FDR adjusted p-values after fitting a Generalized Linear Mixed-Effects Model (GLMM_histopathology).

Supplementary Table 5

Supplementary Table 5c–h are the lists of differentially expressed genes for various comparisons between microglia subclusters and genotypes as identified by the gene-set enrichment analysis (see Methods for details) using the two-sided Wilcoxon Rank Sum test as implemented in the FindMarkers function of the Seurat package.

Supplementary Table 6

Associations between proportion of cell types in 15 microglia subclusters and four histopathological parameters. For each microglia subcluster, the table reports the logOddsRatio for unit change in histopathology, the corresponding standard error, unadjusted p-values, and FDR adjusted p-values after fitting a Generalized Linear Mixed-Effects Model (GLMM_histopathology).
