# Supplementary material for: Neuronal APOE4 removal protects against tau-mediated gliosis, neurodegeneration and myelin deficits
Source: Nat Aging. 2023 Feb 20;3(3):275–96. doi: 10.1038/s43587-023-00368-3 (PMC10154214; doi:10.1038/s43587-023-00368-3)
Supplement: Supplementary file 2 — Reporting Summary [file 43587_2023_368_MOESM2_ESM.pdf]

nature research

Corresponding author(s): Yadong Huang

Last updated by author(s): 01-08-2023

Reporting Summary

Nature Research wishes to improve the reproducibility of the work that we publish. This form provides structure for consistency and transparency in reporting. For further information on Nature Research policies, see our Editorial Policies and the Editorial Policy Checklist.

Statistics

For all statistical analyses, confirm that the following items are present in the figure legend, table legend, main text, or Methods section.

n/a	Confirmed	
☐	☒	The exact sample size (*n*) for each experimental group/condition, given as a discrete number and unit of measurement
☐	☒	A statement on whether measurements were taken from distinct samples or whether the same sample was measured repeatedly
☐	☒	The statistical test(s) used AND whether they are one- or two-sided<br>*Only common tests should be described solely by name; describe more complex techniques in the Methods section.*
☐	☒	A description of all covariates tested
☐	☒	A description of any assumptions or corrections, such as tests of normality and adjustment for multiple comparisons
☐	☒	A full description of the statistical parameters including central tendency (e.g. means) or other basic estimates (e.g. regression coefficient) AND variation (e.g. standard deviation) or associated estimates of uncertainty (e.g. confidence intervals)
☐	☒	For null hypothesis testing, the test statistic (e.g. F, t, r) with confidence intervals, effect sizes, degrees of freedom and P value noted<br>*Give P values as exact values whenever suitable.*
☒	☐	For Bayesian analysis, information on the choice of priors and Markov chain Monte Carlo settings
☒	☐	For hierarchical and complex designs, identification of the appropriate level for tests and full reporting of outcomes
☐	☒	Estimates of effect sizes (e.g. Cohen's *d*, Pearson's *r*), indicating how they were calculated

Our web collection on statistics for biologists contains articles on many of the points above.

Software and code

Policy information about availability of computer code

Data collection	No software was used in the collection of data for this study, aside from the drivers for microscopes and sequencing machines. The specifications for those hardware are described in the Methods section.
Data analysis	Analyses were conducted using the following publicly-available software packages: Seurat v4.0.4, Seurat v4.0.5, Cellranger v3.0.2, Cellranger v6.1.1, glmGamPoi v1.6.0, clusterProfiler v4.2.1, lme4 v.1.1-27.1, factoextra v1.0.7, ggplot2 v3.3.5, dplyr v1.0.7, gridExtra v2.3, magrittr v2.0.1, gdata v2.18.0, org.Mm.eg.db v3.14.0, pheatmap v1.0.12, EnhancedVolcano v1.14.0, R v4.1.0, R v4.1.2, and Fiji v1.0 (ImageJ). Western blot images were analyzed using Image Studio Lite v.5.2.5 (LI-COR). GraphPad Prism v9.2.0 was used for some statistical analyses and graphing.<br><br>All data analysis packages are included in the Code Availability section in the Methods. Packages that were dependencies or used to make figures are also listed in the Code Availability section in the Methods.<br><br>All other data analyses were done with custom R and shell scripting that are available via Zenodo at https://doi.org/10.5281/zenodo.7508271.

For manuscripts utilizing custom algorithms or software that are central to the research but not yet described in published literature, software must be made available to editors and reviewers. We strongly encourage code deposition in a community repository (e.g. GitHub). See the Nature Research guidelines for submitting code & software for further information.

Data

Policy information about availability of data

All manuscripts must include a data availability statement. This statement should provide the following information, where applicable:

- Accession codes, unique identifiers, or web links for publicly available datasets
- A list of figures that have associated raw data
- A description of any restrictions on data availability

The Homo sapiens microtubule associated protein Tau (MAPT) sequence is available at: https://www.ncbi.nlm.nih.gov/nuccore/NM_001123066

The reference mouse genome sequence (GRCm38) from Ensembl (release 98) is available at: http://ftp.ensembl.org/pub/release-98/fasta/mus_musculus/dna/Mus_musculus.GRCm38.dna.primary_assembly.fa.gz

The reference mouse gene annotation file from GENCODE (release M23) is available at: http://ftp.ebi.ac.uk/pub/databases/gencode/Gencode_mouse/release_M23/gencode.vM23.primary_assembly.annotation.gtf.gz

Mouse single-nucleus RNA sequencing datasets generated in association with this study are available in the Gene Expression Omnibus (GEO) under the accession number GSE221215. Source Data associated with Figure 5, 7, and 8 as well as Extended Data Figures 4-8 are available in the Supplementary Information.

The Kyoto Encyclopedia of Genes and Genomes (KEGG) Pathways database is available at: https://www.genome.jp/kegg/pathway.html

Field-specific reporting

Please select the one below that is the best fit for your research. If you are not sure, read the appropriate sections before making your selection.

☒ Life sciences ☐ Behavioural & social sciences ☐ Ecological, evolutionary & environmental sciences

For a reference copy of the document with all sections, see nature.com/documents/nr-reporting-summary-flat.pdf

Life sciences study design

All studies must disclose on these points even when the disclosure is negative.

Sample size

For immunohistochemical, biochemical, and electrophysiological analyses, sample sizes were determined using effect sizes estimated from pilot cohorts and previous studies.

For most immunohistochemical analyses, we utilized n>=15 mice per genotype group to achieve >=80% power to observe differences of >20% between genotype groups with two-sided significance of 0.05. For some immunohistochemical analyses with drastic differences between genotype groups in pilot cohorts, we utilized n=8 mice per genotype group to achieve >=80% power to observe differences of >20% between genotype groups with two-sided significance of 0.05.

For biochemical analyses, we utilized n>=11 mice per genotype as some brains were preserved for snRNA-seq experiments on mice that had already undergone rigorous pathological analyses, sufficient for a power of >=80% to observe differences of >20% between genotype groups with two-sided significance of 0.05.

For slice electrophysiological studies, sample size was determined using pilot experiments, with n>=11 and N>=2 per group sufficient for a power of >=80%.

For single-nucleus RNA-sequencing experiments, sample sizes were determined by a power analysis using effect sizes estimated from our previous studies and a literature search. Nuclei were isolated from 4 mice per mouse genotype to ensure an n of >= 3 mice per genotype, resulting in a total of 12 samples. Sample preparation was successful for 11 out of 12 samples. One sample initially processed on a separate day had low quality and quantity of cDNA recovery due to expired reagents and were excluded from downstream analyses with Seurat. All other 11 samples were prepared with a new batch of sample preparation reagents and had high quality and quantity of cDNA recovery. See methods section for more details.

Data exclusions

No data were excluded.

Replication

The effects of APOE4 versus APOE3 on tau pathology, hippocampal degeneration, astrogliosis, and microgliosis in the same tauopathy mouse model were replicated in a different cohort of mice. The snRNA-seq study was only done in one cohort of mice.

Randomization

Mice were randomly allocated to groups for all immunohistochemical, biochemical, and electrophysiological studies.

For single-nucleus RNA-sequencing studies, the mice had undergone rigorous pathological characterization and we selected mice in each genotype group that represented near the quantified average for all pathological parameters for that genotype group. Due to the variability of pathology in certain genotype groups, specifically selecting mice that are good representatives of each genotype group for sequencing analysis allowed us to make more accurate correlations between pathologies and sequencing data.

Blinding

Investigators were blinded to all mouse genotype groups during data collection and data analyses for all immunohistochemical, biochemical, and electrophysiological studies.

Investigators were not blinded during analysis of the single-nucleus RNA-sequencing datasets, as sample metadata (such as mouse genotype groups) was needed to conduct quality control and comparisons. Additionally, the pathological data for each mouse was required to conduct association analyses.

Reporting for specific materials, systems and methods

We require information from authors about some types of materials, experimental systems and methods used in many studies. Here, indicate whether each material, system or method listed is relevant to your study. If you are not sure if a list item applies to your research, read the appropriate section before selecting a response.

Materials & experimental systems

n/a	Involved in the study
☐	☒ Antibodies
☒	☐ Eukaryotic cell lines
☒	☐ Palaeontology and archaeology
☐	☒ Animals and other organisms
☒	☐ Human research participants
☒	☐ Clinical data
☒	☐ Dual use research of concern

Methods

n/a	Involved in the study
☒	☐ ChIP-seq
☒	☐ Flow cytometry
☒	☐ MRI-based neuroimaging

Antibodies

Antibodies used

Mouse anti-AT8 (1:100), Invitrogen, #MN1020
Rabbit anti-ApoE (1:200), Cell Signaling, #13366S
Mouse anti-Calmodulin (1:100), Thermofisher, #MA3-917
Rat anti-CD68 (1:100), Bio-Rad, #MCA1957
Rabbit anti-Cleaved Caspase-3 (1:100), Cell Signaling, #9661
Rabbit anti-Cre recombinase (1:800), Cell Signaling, #15036S
Mouse anti-GFAP, MilliporeSigma (1:800), #MAB3402
Goat anti-GFAP (1:800), NovusBio, #N100-53809
Mouse anti-GFP (1:5,000), Thermofisher, #A-11120
Rabbit anti-Hsp90 (1:100), NovusBio, #NB120-2928
Mouse anti-HT7 (1:200), Peter Davies, #N/A
Rabbit anti-Iba1 (1:300), Wako, #019-19741
Goat anti-Iba1 (1:100), Abcam, #ab5076
Rabbit anti-MBP (1:500), Abcam, #ab40390
Rat anti-Mertk (1:100), Thermofisher, #14-5751-82
Guinea Pig anti-NeuN (1:500), MilliporeSigma, #ABN90
Rabbit anti-NG2 (1:500), MilliporeSigma, #AB5320
Goat anti-Olig2, R&D Systems (1:100), #AAF2418
Mouse anti-Olig2 (1:100), MilliporeSigma, #MABN50
Rabbit anti-S100β (1:200), Abcam, #ab52642
Rabbit anti-Tmsb4x (1:100), Thermofisher, #PA50584
Rabbit anti-TUJ1 (1:20,000), Biolegend, #802001
Mouse anti-Ubb (1:100), Thermofisher, #13-1600
Donkey anti-mouse Biotin-SP (1:200), Jackson Immuno, #715-065-150
DAPI (1:30,000), Thermofisher, #62248
Donkey anti-mouse 488 (1:1000), Abcam, #ab150105
Donkey anti-rabbit 488 (1:1000), Abcam, #ab150073
Donkey anti-rat 488 (1:1000), Abcam, #ab150153
Donkey anti-mouse 594 (1:1000), Abcam, #ab150108
Donkey anti-rabbit 594 (1:1000), Abcam, #ab150076
Donkey anti-guinea pig 594 (1:1000), Jackson Immuno, #706-585-148
Donkey anti-mouse 647 (1:1000), Abcam, #ab150107
Donkey anti-rabbit 647 (1:1000), Abcam, #ab150075
Donkey anti-guinea pig 647 (1:1000), Jackson Immuno, #706-605-148
Donkey anti-mouse IRDye 800CW (1:20,000), LI-COR, #926-32212
Donkey anti-rabbit IRDye 680RD (1:20,000), LI-COR, #926-68073

Validation

Mouse anti-AT8, validated for IHC and WB in mouse tissue
Rabbit anti-ApoE, valiated for IHC of human ApoE protein in mouse tissue by previous studies
Mouse anti-Calmodulin, validated for IHC in mouse tissue
Rat anti-CD68, validated for IHC in mouse tissue
Rabbit anti-Cleaved Caspase-3, validated for IHC in mouse tissue
Rabbit anti-Cre recombinase, validated for IHC in mouse tissue
Goat anti-GFAP, validated for IHC in mouse tissue
Mouse anti-GFAP, validated for IHC in mouse tissue
Mouse anti-GFP, validated for IHC of GFP tag
Rabbit anti-Hsp90, validating for IHC in mouse tissue
Mouse anti-HT7, validated for IHC in mouse tissue by previous studies

Goat anti-Iba1, validated for IHC in mouse tissue
Rabbit anti-Iba1, validated for IHC in mouse tissue
Rabbit anti-MBP, validated for IHC in mouse tissue by previous studies
Rat anti-Mertk, validated for IHC in mouse tissue
Guinea Pig anti-NeuN, validated for IHC in mouse tissue
Rabbit anti-NG2, validated for IHC in mouse tissue
Goat anti-Olig2, validated for IHC in mouse tissue
Mouse anti-Olig2, validated for IHC in mouse tissue
Rabbit anti-S100β, validated for IHC in mouse tissue
Rabbit anti-Tmsb4x, validated for IHC in mouse tissue
Rabbit anti-TUJ1, validated for WB in mouse tissue
Rabbit anti-Ubb, validated for IHC in mouse tissue
Donkey anti-mouse Biotin-SP, validated for IHC in mouse tissue
DAPI, validated for IHC in mouse tissue
Donkey anti-mouse 488, validated for IHC in mouse tissue
Donkey anti-rabbit 488, validated for IHC in mouse tissue
Donkey anti-rat 488, validated for IHC in mouse tissue
Donkey anti-mouse 594, validated for IHC in mouse tissue
Donkey anti-rabbit 594, validated for IHC in mouse tissue
Donkey anti-guinea pig 594, validated for IHC in mouse tissue
Donkey anti-mouse 647, validated for IHC in mouse tissue
Donkey anti-rabbit 647, validated for IHC in mouse tissue
Donkey anti-guinea pig 647, validated for IHC in mouse tissue
Donkey anti-mouse IRDye 800CW, validated for WB in mouse tissue
Donkey anti-rabbit IRDye 680RD, validated for WB in mouse tissue

Animals and other organisms

Policy information about studies involving animals; ARRIVE guidelines recommended for reporting animal research

Laboratory animals

ApoE3-fKI: Apoetm2(APOE_i3)Yhg, available from Yadong Huang (Bien-Ly N, Gillespie AK, Walker D, Yoon SY, Huang Y. Reducing human apolipoprotein E levels attenuates age-dependent Aβ accumulation in mutant human amyloid precursor protein transgenic mice. J. Neurosci. 2012 Apr. 4; 32(14)4802-11).

ApoE4-fKI: Apoetm3(APOE_i4)Yhg, available from Yadong Huang (Bien-Ly N, Gillespie AK, Walker D, Yoon SY, Huang Y. Reducing human apolipoprotein E levels attenuates age-dependent Aβ accumulation in mutant human amyloid precursor protein transgenic mice. J. Neurosci. 2012 Apr. 4; 32(14)4802-11).

Syn1-Cre: B6.Cg-Tg(Syn1-Cre)671Jxm/J, The Jackson Laboratory, #003966
Tau-P301S (PS19): B6;C3-Tg(Prnp-MAPT*P301S)PS19Vle/J, The Jackson Laboratory, #008169

ApoE4-fKI and ApoE3-fKI mice were previously crossed with Syn1-Cre (#003966) mice to generate fE/Syn1-Cre mosue line (Knoferle J, Yoon SY, Walker D, Leung L, Gillsepie AK, Tong LM, Bien-Ly N, Huang Y. Apolipoprotein E4 produced in GABAergic interneurons causes learning and memory deficits in mice. J. Neurosci. 2014 Oct. 15; 34(42)1469-14078).

The fE/Syn1-Cre mice were then crossed to Tau-P301S (PS19 line) (#008169) mice to generate PS19-fE mice with no Cre or Syn1-Cre. Both male and female mice were used for pathological, biochemical, and electrophysiological analysis. Mice were analyzed at 10-months-old.

Wild animals

No wild animals were used in this study.

Field-collected samples

No field-collected samples were used in this study.

Ethics oversight

All animal experiments were conducted in accordance with the guidelines and regulation of the National Institutes of Health, the University of California, and the Gladstone Institutes under the protocol AN176773. All protocols and procedures followed the guidelines of the Laboratory Animal Resource Center at the University of California, San Francisco (UCSF) and the ethical approval of the UCSF IACUC.

Note that full information on the approval of the study protocol must also be provided in the manuscript.
