# Supplementary figures and images for: Neuronal APOE4 removal protects against tau-mediated gliosis, neurodegeneration and myelin deficits
Source: Nat Aging. 2023 Feb 20;3(3):275–96. doi: 10.1038/s43587-023-00368-3 (PMC10154214; doi:10.1038/s43587-023-00368-3)

Figure 1

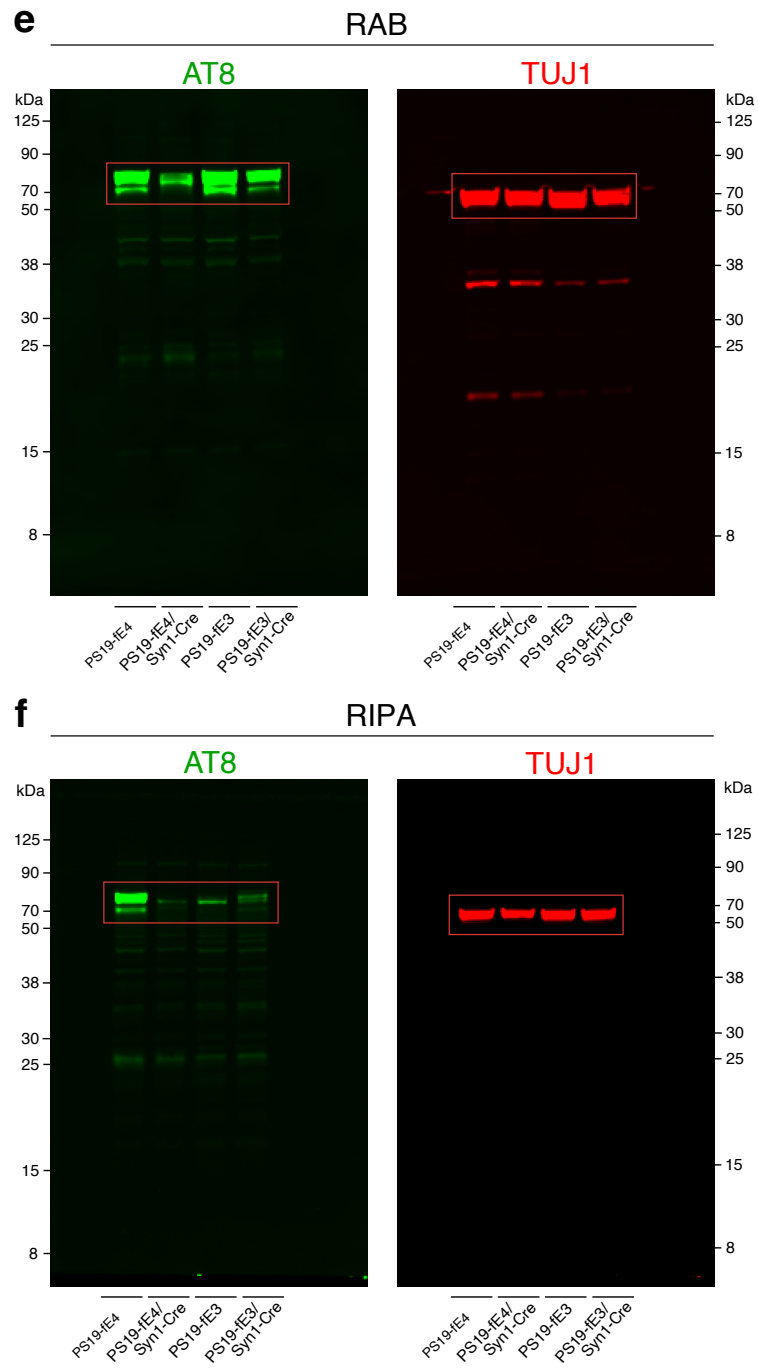

Supplement: Source Data Fig. 1 — Uncropped scans of western blot gel image source data for Fig. 1e,f. [file 43587_2023_368_MOESM10_ESM.pdf]
